# Spatial Isoforms Reveal the Mechanisms of Metastasis

**DOI:** 10.1002/advs.202402242

**Published:** 2024-09-23

**Authors:** Yin Yin, Yuhao Wang, Xiao Yu, Yang Li, Yahui Zhao, Yanfeng Wang, Zhihua Liu

**Affiliations:** ^1^ State Key Laboratory of Molecular Oncology National Cancer Center National Clinical Research Center for Cancer Cancer Hospital Chinese Academy of Medical Sciences and Peking Union Medical College Beijing 100021 China; ^2^ Department of Comprehensive Oncology National Cancer Center/National Clinical Research Center for Cancer Cancer Hospital Chinese Academy of Medical Sciences and Peking Union Medical College Beijing 100021 China; ^3^ Institute of Cancer Research Henan Academy of Innovations in Medical Sciences Zhengzhou Henan 450000 China

**Keywords:** CD74, chemoresistant, esophageal squamous cell carcinoma, exhausted T cells, metastasis, spatial isoforms, tumor‐associated macrophage

## Abstract

In esophageal squamous cell carcinoma (ESCC), lymph node (LN) metastasis is associated with poor survival. Emerging evidence has demonstrated elevated *CD8* ^+^ T‐cell levels in metastatic LNs following immunotherapy and increased chemoresistance. However, the underlying regulatory mechanisms of *CD8* ^+^ T cells in chemoresistant/metastatic patients have not been elucidated. Given that metastasis is linked to aberrant splicing patterns, transcripts with alternative splicing forms also play a critical role. With spatial transcriptomics (ST), spatial isoform transcriptomics (SiT), single‐cell RNA sequencing (scRNA‐seq), and T‐cell receptor (TCR) sequencing, the spatial isoforms are analyzed quantitatively in human solid tumors and LNs. These isoforms are classified according to expression and spatial distribution patterns and proposed that the temporal heterogeneity in isoforms is attributed to isoform biogenesis dynamics. *C1QC* ^+^ tumor‐associated macrophages (TAMs) contribute to the formation of metastases in the context of both immunotherapy and chemotherapy. Additionally, *CD74* isoforms can be targeted by mRNA drugs, such as antisense oligonucleotides (ASOs) and RNA interference (RNAi), to prevent chemoresistance and metastasis. Overall, this work suggests that *C1QC* ^+^ TAMs interact with *CD8* ^+^ *CXCL13* ^+^ Tex cells via enrichment with the *CD74* isoform in the ESCC ‘s metastatic lymph node.

## Graphical abstract

By using Spatial Isoform Transcriptomics (SiT), *CD74* isoforms are identified for their close relationship with the enrichment of *C1QC* ^+^ TAMs and *CD8^+^ CXCL13^+^* Tex in the metastatic lymph node of ESCC. In pan‐epithelial cancers, *C1QC* ^+^ TAMs suppress the cytotoxicity of *CD8^+^ CXCL13^+^* Tex cells through the MIF–CD74 ligand‐receptor pair.

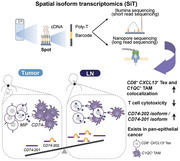

## 1 Introduction

Esophageal squamous cell carcinoma (ESCC) is characterized by oncogenic splicing events.^[^ ^1^, ^2^, ^3^ ^]^ Esophageal cancer (EC) was the seventh most common cancer worldwide and the sixth leading cause of cancer death in 2020.^[^ ^4^ ^]^ EC can be classified into two subtypes: esophageal adenocarcinoma and ESCC. ESCC accounts for 85% of EC cases.^[^ ^5^ ^]^ The 5‐year survival rate of ESCC patients is nearly 10–15%, and ≈50% of ESCC cases occur in China. In recent studies, *CD8* ^+^ *CXCL13* ^+^ exhausted T (Tex) cells have been reported to be predictors of immune checkpoint blockage (ICB) therapy response. A meta‐analysis correlated *CD8* ^+^ *CXCL13* ^+^ T cells with favorable ICB outcomes.^[^ ^6^ ^]^ The *CXCL13* ^+^ T‐cell abundance increases after combination therapy and decreases after therapy with paclitaxel alone.^[^ ^7^ ^]^ Moreover, as the most abundant immune population in the tumor microenvironment (TME), tumor‐associated macrophages (TAMs) suppress the activities of cytotoxic T cells at both primary and metastatic sites.^[^ ^8^, ^9^ ^]^ Through losing the protective function of homeostasis, TAMs arise from tissue‐resident macrophages (TRMs) localized at a tumor site and from bone marrow (BM)‐derived monocytes that are recruited to tumors. TAMs are highly heterogeneous, and the significance and therapeutic potential of their diversity are evolving.

However, these studies did not include ESCC patients and did not examine the regulatory role of alternative splicing (AS). Investigating the TME spatially could help elucidate the mechanisms linking chemoresistance and metastasis, particularly in the lymph node (LN), to guide optimal ESCC treatment strategies. As an emerging field of drug discovery, AS is being targeted as a major therapeutic strategy.^[^ ^10^ ^]^ Several hallmarks of cancer initiation and metastasis, such as unlimited proliferation, evasion of growth‐suppressing signals, dysregulated metabolic processes, and cellular phenotypes, are associated with aberrant splicing patterns.^[^ ^11^ ^]^ Although previous studies have comprehensively profiled the landscape of AS in ESCC, the spatial distribution of isoforms is unclear.^[^ ^12^ ^]^ With the emergence of single‐cell RNA sequencing (scRNA‐seq) and spatial transcriptomics, the relationships between expression heterogeneity across various cell types, states and locations, and oncogenesis have been easily resolved.^[^ ^13^, ^14^, ^15^ ^]^ However, comprehensive analysis of splice variation via short‐read sequencing is limited; although exon junctions can be observed, identifying the resolution of full‐length isoforms is extremely challenging. Recently, several studies have revealed the characteristics of isoforms and identified differential isoform usage by combining scRNA‐seq with spatial transcriptomic (10X) long‐read sequencing technology (PacBio or Oxford Nanopore sequencing (ONT)).^[^ ^16^, ^17^, ^18^, ^19^ ^]^ Spatial isoform transcriptomics (SiT) is an unbiased method based on spatial in situ capture used to detect and quantify the spatial expression of splicing variants through combination with third‐generation sequencing.^[^ ^19^ ^]^

Our study utilized SiT, scRNA‐seq, and T‐cell receptor (TCR) analyses of tumor and LN tissues to demonstrate that the temporal heterogeneity of the spatial *CD74* isoform correlates with the expanded enrichment of *C1QC* ^+^ TAMs and *CD8* ^+^ *CXCL13* ^+^ Tex cells in LN metastasis of ESCC. We hypothesized that the thyroglobulin type‐1 domain, which alters the secondary structure and solvent accessibility, promotes the enrichment of *C1QC* ^+^ TAMs and *CD8* ^+^ *CXCL13* ^+^ T cells. Moreover, we revealed that *C1QC* ^+^ TAMs potentially inhibit the cytotoxicity of *CD8* ^+^ *CXCL13* ^+^ Tex cells via the MIF‐CD74 ligand‐receptor pair. Furthermore, targeting the *CD74* isoform could potentially disrupt this interaction, thereby preventing metastasis and chemoresistance in ESCC.

## 2 Results

### 2.1 Characterization of Isoform Expression Patterns in ESCC

**Figure 1:**
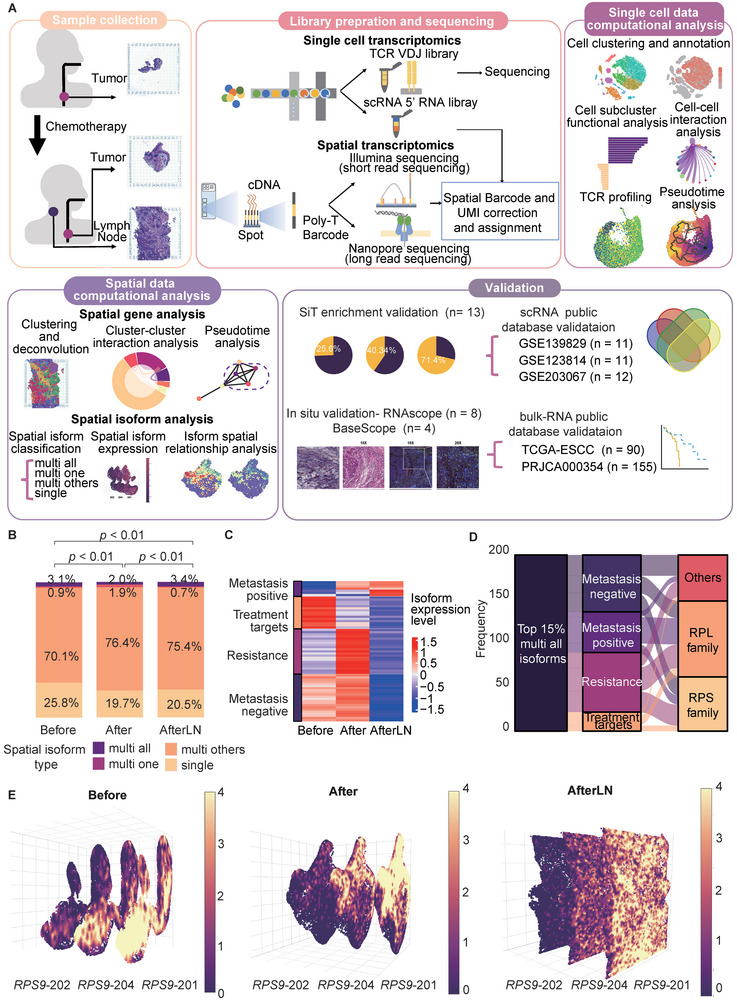
Overview of workflow and spatial isoform information. A) Illustration of the overall workflow. B) Overview of the spatial isoform class distribution in each sample. *P* value was calculated by a chi‐square test, n (Before) = 21 413, n (After) = 32 618, n (AfterLN) = 33 246. C) Heatmap of isoform expression in each sample. The left side indicates the potential clinical marker types of the isoforms. D) Sankey diagram of the top 15% most highly expressed “multi all” isoforms. The flows show the isoform distribution across the three isoform characteristics. The colors indicate the potential clinical marker types of the isoforms. E) *RPS9* isoform spatial distribution and expression level in each sample. All isoforms were stacked in a 3D format. The colors indicate the expression levels of the isoforms.

To study the relationship between the isoform level and chemotherapy response, we designed a similar workflow to compare the spatial transcriptomics data of the Illumina sequencing and ONT sequencing methods (**Figure** **1**A). We focused on three samples from one patient: a tumor sample before chemotherapy (Before), a tumor sample after chemotherapy (After), and a lymph node sample after chemotherapy (AfterLN). All three samples were subjected to single‐cell sequencing with TCR. Based on a previous SiT workflow, full‐length 10X Genomics Visium cDNA libraries were split for Illumina sequencing and ONT sequencing.^[^ ^19^ ^]^ We generated 320–410 million reads per sample from Illumina sequencing and 45–83 million reads per sample from ONT sequencing (Table S1, Supporting Information). The average read quality and read length of all the samples were similar (Figure S1A, Supporting information). The majority of the sequences of all samples had a quality score from 10 –16. The highest sequencing saturation in the three samples was >90% (Figure S1B, Supporting Information). The maximum read length of the three samples reached 338 kb, and the average read length ranged from 753 bp to 970 bp. With the aid of short‐read sequencing data, UMI, and spatial barcode assignment were performed for long‐read sequencing.^[^ ^19^ ^]^ Post‐correction of barcodes and UMIs, we retained ≈46% of the raw reads (Figure S1E, Supporting Information). Importantly, the Pearson correlation for both UMI and gene levels exceeded 0.93 across all three samples (Figure S2A, Supporting Information). Our comprehensive analysis unveiled a rich landscape of isoforms: a total of 87277 unique isoforms originating from 59700 genes were identified in the three samples (Table S1, Supporting Information). Additionally, a mean of 763 isoforms per spatially barcoded spot were observed in all three samples (Table S1, Supporting Information). Notably, the AfterLN sample exhibited the highest density of isoforms per spot (Figure S1C,D,F, Supporting Information), suggesting distinct molecular heterogeneity in this lymph node specimen (Figure S1C,D,F, Supporting Information). Spatially clustered regions were termed niches. We compared the niche clustering patterns derived from gene‐level short‐read data with those obtained from isoform‐level long‐read data. Clustering from gene‐level short‐read data was similar to clustering from isoform‐level long‐read data (Figure S2B,C, Supporting Information). All niche similarities of samples were from 58.7% to 76.3%.

To achieve a comprehensive understanding of isoform dynamics, we initiated a systematic classification based on the relationship between isoforms and their corresponding genes, as well as their spatial distribution patterns: 1) multi‐all: isoforms with various “alternate isoforms” for a corresponding gene that are distributed to all spatial niches; 2) multi one: isoforms with various “alternate isoforms” for a corresponding gene that are uniquely expressed in one spatial niche; 3) multi others: isoforms with various “alternate isoforms” for a corresponding gene that are distributed to two or more spatial niches but not all spatial niches; and 4) single: an isoform that is the only isoform of a corresponding gene. In all the samples, “single” isoforms represented ≈25% of all the isoforms. Additionally, most of the isoforms were categorized as “multi others” (Figure 1B). Next, we classified the isoforms according to their expression pattern: 1) metastasis negative: expression decreased in metastatic LNs; 2) metastasis positive: expression increased in metastatic LNs; 3) resistance: expression in tumors increased after chemotherapy; and 4) treatment target: expression in tumors decreased after chemotherapy.

Among all the isoforms of the Before sample, nearly 25% were “single” isoforms, while 75% were “multi all”, “multi one”, and “multi others”. Furthermore, ≈0.9% of the isoforms were distributed uniquely in only one niche (Figure 1B). However, after the chemotherapy, both After and AfterLN samples decreased their “single” isoform percentage. Our findings shed light on the alternative splicing level within an ESCC tumor and lymph node (LN) after chemotherapy.

Among the 15% most highly expressed “multi all” isoforms, we identified isoforms with all four different expression patterns (Figure 1C). Notably, these isoforms predominantly originate from the small ribosomal subunit (RPS) and large ribosomal subunit (RPL) families (Figure 1D). Our attention was drawn to the *RPS9* isoforms (*RPS9*‐201, *RPS9*‐202, *RPS9*‐204), which consistently exhibit increased expression levels across all samples (Figure 1E; Figure S3, Supporting Information). Since alternative RNA splicing is known to be involved in the regulation of ribosomal functions,^[^ ^20^ ^]^ taken together, these findings and our observations of *RPS9* isoform structures suggested that there is temporal heterogeneity in ribosomal biogenesis that leads to increased expression of the isoforms.

Collectively, by applying SiT, the characterization of isoform expression patterns in ESCC was achieved. We can track isoform variations at the near‐cellular level, unraveling their spatial distribution and functional roles. Our findings underscore the importance of SiT for its power to explore the dynamic nature of carcinogenesis and metastasis, with implications for cellular adaptation and response to chemotherapy.

### 2.2 *C1QC* ^+^ TAMs Exert Cytotoxicity Suppression on *CD8^+^ CXCL13^+^* Tex Cells through the MIF‐CD74 Ligand‐Receptor Pair

**Figure 2:**
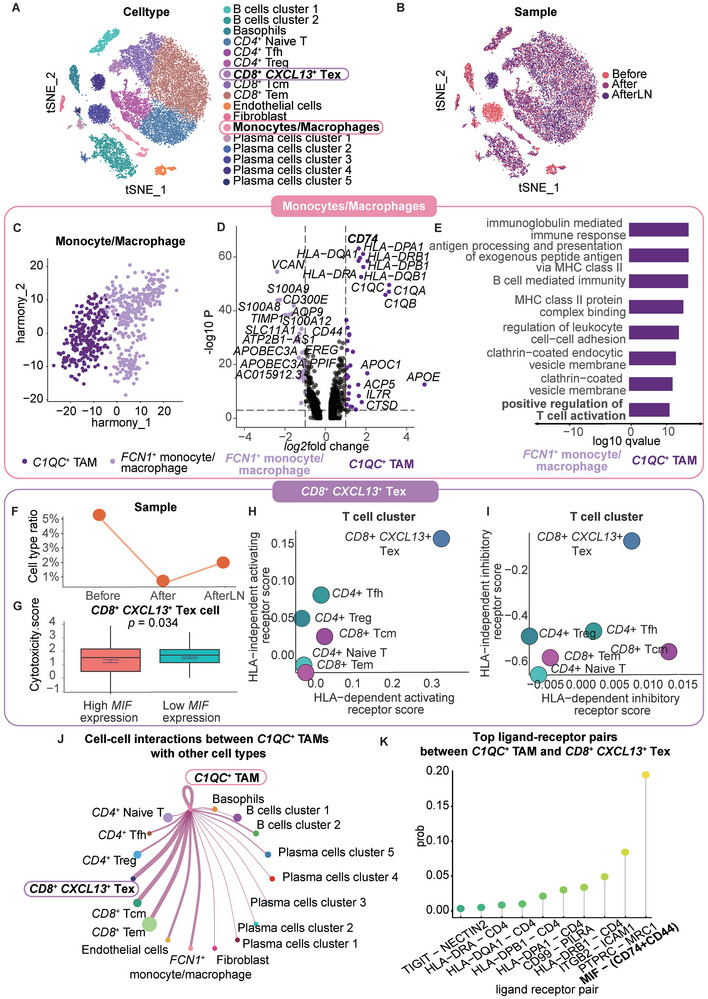
*C1QC^+^* TAMs suppress the cytotoxicity of *CD8^+^ CXCL13^+^* Tex cells through the MIF‐CD74 ligand‐receptor pair. A) TSNE of cell type clustering of single‐cell transcriptomic data. B) TSNE of sample clustering of single‐cell transcriptomic data. C) UMAP plot of the cell type clustering of TAMs after Harmony analysis. The isoform spatial distribution and expression level in each sample are shown. The colors indicate the expression levels of the isoforms. D) Volcano plot of genes with differential expression between *C1QC* ^+^ TAMs and *FCN1^+^* TAMs. Top 15 genes with the highest log_2_fold change for both cell types are labeled. CD74 is bolded. E) Gene Ontology pathway enrichment of genes with differential expression between *C1QC^+^* TAMs and *FCN1* ^+^ TAMs. The colors indicate the cell types. Important pathways are shown in bold. F) *CD8^+^ CXCL13^+^* Tex cell ratio in each sample. G) Boxplot representing the cytotoxicity GSVA score between *CD8^+^ CXCL13^+^* Tex cells with high *MIF* expression (> mean *MIF* expression) and low MIF expression (< mean *MIF* expression) in *CD8^+^ CXCL13^+^* Tex cells. *P* value was calculated by an unpaired two‐tailed Student's t‐test, n (high *MIF* expression) = 260, n (low *MIF* expression) = 153. H) Dot plot representing the HLA‐independent activating receptor score and the HLA‐dependent activating receptor score for each T‐cell type. I) Dot plot representing the HLA‐independent inhibitory receptor score and the HLA‐dependent inhibitory receptor score for each T‐cell type. J) Cell crosstalk diagram for all cell types. The width of the line indicates the strength of the interaction. The size of each cell type dot indicates the cell number. The color of each cell type dot indicates the cell type. K) Lollipop plot of the top 10 ligand‐receptor pairs between *CD8^+^ CXCL13^+^* Tex cells and *C1QC^+^* TAMs. The colors indicate the ligand‐receptor pairs.

To elucidate the cellular dynamics during ESCC metastasis and the pre‐ and posttreatment periods, we cataloged all the cells into seventeen cell lineages. Based on the canonical marker data, two B‐cell clusters, basophils, *CD4* ^+^ naïve T cells, *CD4* ^+^ Tfhs, CD4^+^ Tregs, *CD8* ^+^ *CXCL13* ^+^ Tex cells, *CD8* ^+^ Tcms, *CD8* ^+^ Tems, endothelial cells, fibroblasts, monocytes/macrophages, and five plasma cell clusters were identified (**Figure** **2**A; Figure S4A, Table S4, Supporting Information). Plasma cell cluster 3 was expressed only in the tumor sample after chemotherapy, while plasma cell clusters 4 and 5 were expressed only in the lymph node sample after chemotherapy (Figure 2B). Additionally, we found that >10% of the cells were monocytes/macrophages before chemotherapy, while the percentage of monocytes/macrophages decreased to <5% after chemotherapy (Figure S4B, Supporting Information).

Since TAMs have been reported to suppress cytotoxic T cells, we further clustered monocytes/macrophages into *C1QC* ^+^ TAMs and *FCN1* ^+^ other monocytes/macrophages based on the canonical markers (Figure 2C; Figure S5A,B, Supporting Information).^[^ ^8^ ^]^ After differential gene expression analysis between *C1QC* ^+^ TAMs and *FCN1* ^+^ monocytes/macrophages, in addition to clustering markers, such as *C1QC*, *C1QB* and *C1QA*, other genes, such as *CD74*, *APOE*, *HLA‐DRA1*, *HLA‐DRB1*, *HLA‐DPB1* and *HLA‐DQB1*, were significantly upregulated in *C1QC* ^+^ TAMs (Figure 2D). Notably, we explored whether the positive regulation of T‐cell activation and antigen processing and the presentation of exogenous peptide antigens via MHC class II were two significantly upregulated pathways (Figure 2E).

Next, we examined the function and ratio of T cells. Among all T‐cell subtypes, *CD8* ^+^ *CXCL13* ^+^ Tex cells expressed the cytotoxicity‐related genes *GZMA*, *GNLY*, *PRF1*, *GZMB*, and *NKG7*. *CD8* ^+^ *CXCL13* ^+^ Tex cells also expressed the exhaustion markers *LAG3*, *TIGIT*, *PDCD1*, *HAVCR2*, and *CTLA4* (Figure S6A, Supporting Information). In addition, *CD8* ^+^ *CXCL13* ^+^ Tex cells expressed high levels of *CXCL13*. Compared with those in other T cells, the genes expressed in CD4^+^ Tfh cells were more highly enriched in proliferation‐related pathways, such as those related to DNA repair, E2F targets, and the G2/M checkpoint. *CD8* ^+^ *CXCL13* ^+^ Tex cells expressed higher levels of proteins in the Notch signaling pathway and WNT‐beta‐catenin signaling pathway after chemotherapy (Figure S6B, Supporting Information). Then, we compared the ratio of *CD8* ^+^ *CXCL13* ^+^ Tex cells among the three samples. The ratio of *CD8* ^+^ *CXCL13* ^+^ Tex cells decreased after chemotherapy (Figure 2F).

We next evaluated the effect of cell‒cell communication on the function of *CD8* ^+^ *CXCL13* ^+^ Tex cells. As a critical communicator in both the extracellular and intracellular compartments, macrophage migration inhibitory factor (*MIF*) can initiate cellular proliferation and prostaglandin E2 production through intracellular transduction cascades after signaling with *CD74*.^[^ ^21^ ^]^ Additionally, *MIF* has been shown to promote T‐cell exhaustion regulation.^[^ ^22^ ^]^ To validate the impairment of *MIF* expression, we then compared the cytotoxicity of *CD8* ^+^ *CXCL13* ^+^ Tex cells in high‐*MIF*‐expressing and low‐*MIF*‐expressing cells. Our findings revealed that *CD8^+^ CXCL13^+^* Tex cells exhibited diminished cytotoxicity when MIF expression levels were elevated (Figure 2G). Additionally, *CD8* ^+^ *CXCL13* ^+^ Tex cells had the highest score in the human leukocyte antigen (HLA)‐independent activating receptor pathway, HLA‐dependent activating receptor pathway, and HLA‐independent inhibitory receptor pathway, implying *CD8^+^ CXCL13^+^* Tex cells may tightly interact with antigen‐presenting cells (APCs) such as TAMs (Figure 2H,I). Therefore, we performed cell‐cell interaction analysis and found that *C1QC^+^* TAM strongly interacted with *CD8^+^ CXCL13^+^* Tex. Accordingly, we assumed that cell‐cell communication may play a key role in the function of *CD8* ^+^ *CXCL13* ^+^ Tex cells and that high cell‐cell communication through *MIF* may impair the cytotoxicity of *CD8* ^+^ *CXCL13* ^+^ Tex cells.

By exploring the ligand‒receptor interactions and cell‒cell interactions between each cluster, we confirmed that *C1QC* ^+^ TAMs strongly interacted with *CD8* ^+^ *CXCL13* ^+^ Tex cells (Figure 2J). Among all ligand‐receptor interactions between *C1QC* ^+^ TAMs and *CD8* ^+^ *CXCL13* ^+^ Tex cells, MIF‐ (CD74+CD44) was the first‐ranked interaction (Figure 2K).

In summary, our investigation highlights the pivotal role of the MIF‐CD74 ligand‐receptor interaction in shaping the tumor microenvironment. *C1QC* ^+^ TAMs could suppress the antitumor response by decreasing the cytotoxicity of T cells.

### 2.3 Metastatic Lymph Nodes Exhibit Expansive Enrichment of T Cells and Macrophages Through Multiple Centers

**Figure 3:**
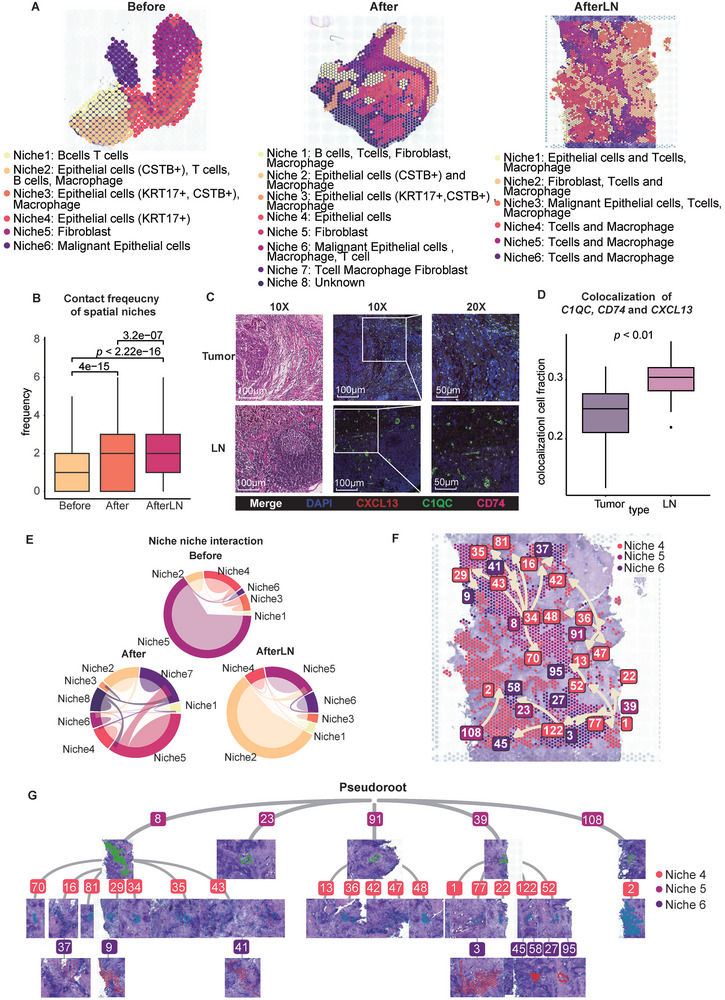
Metastatic lymph nodes in ESCC exhibit expansive enrichment of T cells and macrophages. A) Spatial transcriptomic spots of all three samples: Before, After, and AfterLN. The colors indicate the niches of each sample. B) Boxplot of the contact frequency of spatial niches in each sample. *P* value was calculated by an unpaired two‐tailed Student's t‐test, n (Before) = 21 413, n (After) = 32 618, n (AfterLN) = 33 246. C) *CD74, C1QC*, and *CXCL13* expression were labeled by RNAscope in situ hybridization. Left: HE; Middle: 10X RNAscope; Right: 20X RNAscope. DAPI: blue, *C1QC*: green, *CD74*: pink, *CXCL13*: red, Merge: white. D) Boxplot of the colocalization of the cell fraction with *CD74*, *C1QC*, and *CXCL13* expression between the tumor and LN samples when the enrichment calculation cutoff is 0.6. *P* value was calculated by an unpaired two‐tailed Student's t‐test. E) Circular plot of the cell‐cell interactions between niches in the three samples. The colors indicate the niche. The area of each bond indicates the frequency of interaction. F) Spatial trajectory of subclusters in niches 4–6. Arrows show the direction of the trajectory. G) Tree plot of the trajectory of subclusters in niches 4–6.

To fully understand the spatial niches and cell distributions in tumors and metastatic LNs, we classified all spatial transcriptomic spots into different spatial niches (**Figure** **3**A; Figure S7A, Supporting Information). We assigned the cell types to each niche through deconvolution by using corresponding single‐cell transcriptomics and ESCC single‐cell data from Xiannian et al.^[^ ^23^ ^]^ By calculating the number of different spatial niches that one spatial spot has in the nearest 6 neighboring spatial spots, we achieved the statistical analysis on the contact frequency of each sample. Our results showed that the contact frequency between all niches was low in the tumor sample before chemotherapy while spatial niches kept a high level of contact frequency in the tumor and lymph node after chemotherapy (Figure 3B).

To determine whether spatial niches exhibited similar expression patterns and cell ratios, we next found that niche 6 in the Before sample, niche 6 in the After sample, and niche 3 in the AfterLN sample exhibited similar epithelial cell expression patterns (Figure S7B, Supporting Information). Moreover, the malignant marker *EPCAM* was highly expressed in these three niches (Figure S7B, Supporting Information).

To assess the suppressive effect of chemotherapy on malignant epithelial cells, we analyzed the copy number variation (CNV) events in the malignant epithelial cells in each sample (Figure S8A, Supporting Information). Similarly, the levels of amplification and deletion of malignant epithelial cells decreased after chemotherapy in the ESCC tumor. However, the amplification and deletion levels of malignant epithelial cells in the LN were similar to pre‐treatment levels. These results confirmed that chemotherapy is effective at inhibiting a high level of CNV events in ESCC tumors but not in chemoresistant LNs.

After confirming the presence of malignant epithelial spatial niches, we found that the ratio of malignant epithelial cells was similar in all the samples (Figure S8B, Supporting Information). However, immune cells (*CD3D* ^+^) existed in all spatial spots in the AfterLN sample, while <75% of the spatial spots had immune cells in the Before and After samples (Figure S8C, Supporting Information).

Furthermore, to explore metastasis features in detail, we constructed a set of metastasis‐associated genes (MAGs), which included *FTL*, *TMBIM6*, *CALD1*, *ITGB1*, *DSTN*, *FABP4*, *ACTB*, *HSP90AA1*, *KRT8*, *RDH10*, *KRT7*, *HNRNPA2B1*, *HSPG1*, *HSPA1A*, *TPM2*, *TLN2*, *PLEC*, *MX1*, *HSPA1B*, *ATP2B4* and *CSRP1* (Figure S9A, Supporting Information). To validate the classification power of the MAG set, we performed gene set enrichment analysis (GSEA) on the HRA003107 dataset.^[^ ^24^ ^]^ The MAG scores calculated by GSEA showed significant differences between the two groups of ESCC patients with different metastatic conditions; specifically, metastatic ESCC patients had higher MAG scores (Figure S9B, Supporting Information). In addition, the overall survival (OS) of patients in the high‐MAG‐score group and the low‐MAG‐score group significantly differed. ESCC patients with higher MAG scores had lower survival probabilities (Figure S9C, Supporting Information). These genes were highly related to cell‐substrate junctions and focal adhesion (Figure S9D, Supporting Information).

To further investigate the enrichment of *CD8^+^ CXCL13^+^* Tex cells and *C1QC* ^+^ TAMs, we performed RNAscope in situ hybridization for the *CD74*, *C1QC*, and *CXCL13* mRNAs (Figure 3C). In both the LN and tumor, colocalization of the *CD74*, *C1QC*, and *CXCL13* mRNAs was observed (Figure 3D).

To evaluate spatial niche crosstalk, we evaluated niche‐niche interactions (Figure 3E; Figure S9E–J, Supporting Information). Niche frequency and niche‐niche interactions had no direct relationship. The niches with the most niche‐niche interactions in each sample included fibroblasts (Figure S9E,G,I, Supporting Information). In metastatic LNs, most ligand‒receptor pairs are related to the collagen family, which includes genes such as *COL1A1*, *COL1A2*, and *COL3A1*, which are markers of cancer‐associated fibroblasts (CAFs). Collagen signatures have been proven to be useful for metastasis prediction.^[^ ^25^, ^26^ ^]^ Additionally, *ITGB1* was one of the identified MAGs. Then, we further determined the spatial distribution of the COL1A1‐ITGB1 pair in the three samples. After chemotherapy, COL1A1‐ITGB1 interactions in the tumor and LN began to occur in T‐cell‐ and macrophage‐containing niches (Figure S10, Supporting Information).

Since the number of niches, including T cells and macrophages, increased in the LN, we further investigated the spatial trajectory of all niches in the AfterLN sample. Since niches 4, 5, and 6 contained both macrophages and T cells, we inspected the spatial trajectory among niches 4, 5, and 6. We found that niche 4 is the pseudoroot of all T‐cell‐included niches and that niche 4 started to transition to niche 5 and niche 6 (Figure 3F). Additionally, upon macrophage enrichment, T cells started to expand and differentiate from multiple centers in the AfterLN sample (Figure 3G). These findings support that the enrichment of *CD8* ^+^ *CXCL13* ^+^ Tex cells and *C1QC* ^+^ TAMs in the LN not only occurs more often than in the tumor but also expands through multiple start points within the LN.

In brief, tumors and LNs exhibited similarities and differences after chemotherapy in spatial TME. Chemotherapy demonstrates efficacy in suppressing a substantial number of CNV events within ESCC tumors. However, its impact on chemoresistant LNs remains limited. In addition, interactions between COL1A1 and ITGB1 commence within specific niches containing both T cells and macrophages in tumors and LNs. Furthermore, metastatic lymph nodes exhibit extensive enrichment of T cells and macrophages across multiple centers.

### 2.4 The Elevated *CD74* Isoform Ratio Signifies the Enrichment of *CD8* ^+^ *CXCL13* ^+^ Tex cells and *C1QC* ^+^ TAMs

**Figure 4:**
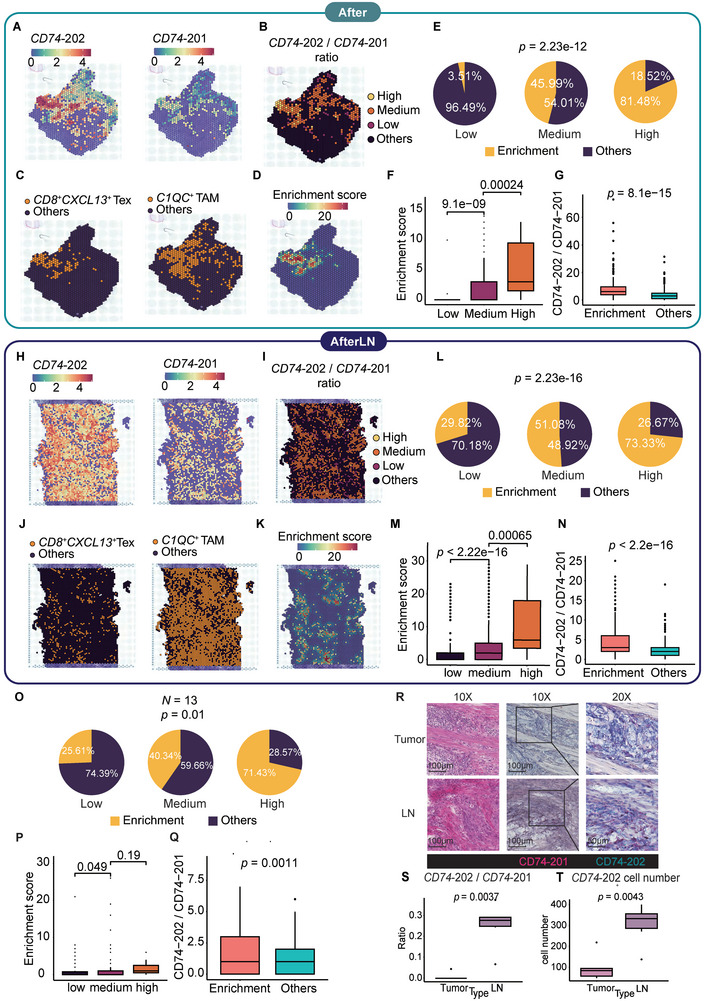
The *CD74* isoform ratio revealed enrichment of *CD8^+^ CXCL13^+^* T cells and *C1QC^+^* TAMs. A,H) Spatial distribution of the *CD74‐*201 isoform and *CD74*‐202 isoform in the After and AfterLN samples. The colors indicate the expression levels. B,I) Spatial distribution of the ratio of *CD74*‐202 isoform expression/*CD74*‐201 isoform expression in the After and AfterLN samples. The color level indicates the ratio of *CD74*‐202 /*CD74*‐201 isoform expression. C,J) Spatial distribution of *C1QC^+^* TAMs, *CD8^+^ CXCL13^+^* Tex cells in the After and AfterLN samples. D,K) *C1QC^+^* TAMs and *CD8^+^ CXCL13^+^* Tex cells’ enrichment score distribution in the After and AfterLN samples. E,L) Pie charts of *C1QC^+^* TAMs and *CD8^+^ CXCL13^+^* Tex cells enriched in low, medium, and high *CD74*‐202/*CD74*‐201 ratio groups in the After and AfterLN samples. *P* values were calculated by a chi‐square test. In the After sample, n (low) = 57, n (medium) = 237, n (high) = 27. In the AfterLN sample, n (low) = 324, n (medium) = 969, n (high) = 15. F,M) Boxplots of enrichment scores in low, medium, and high *CD74*‐202 isoform expression/*CD74*‐201 ratio groups in the After and AfterLN samples. *P* value was calculated by an unpaired two‐tailed Student's t‐test G,N) Boxplots of *CD74*‐202 /*CD74*‐201 ratio in enrichment spots (enrichment score > 0) and other spots in the After and AfterLN samples. *P* value was calculated by an unpaired two‐tailed Student's t‐test. O) Pie charts of *C1QC^+^* TAMs and *CD8^+^ CXCL13^+^* Tex cells enriched in low, medium, and high *CD74*‐202/*CD74*‐201 ratio groups in the validation samples. *P* value was calculated by a chi‐square test, n (low) = 82, n (medium) = 199, n (high) = 7. P) Boxplots of enrichment scores in low, medium, and high *CD74*‐202 isoform expression/*CD74*‐201 ratio groups in the validation samples. *P* value was calculated by an unpaired two‐tailed Student's t‐test. Q) Boxplots of *CD74*‐202 /*CD74*‐201 ratio in enrichment spots (enrichment score > 0) and other spots in the validation samples. *P* value was calculated by an unpaired two‐tailed Student's t‐test. R) Detection of the *CD74‐*202 and *CD74‐*201 isoforms in the tumor and LN samples labeled by BaseScope hybridization technology. Left: HE; middle: 10X BaseScope; right: 20X BaseScope. Dark purple elements indicate counterstained nuclei. *CD74‐*201: pink, *CD74‐*202: turquoise. S) Boxplot of the ratio of *CD74‐*202 to *CD74‐*201 between tumors and LNs. *P* value was calculated by an unpaired two‐tailed Student's t‐test. T) Boxplot of the *CD74‐*202 count between tumors and LNs. *P* value was calculated by an unpaired two‐tailed Student's t‐test.

To elucidate the role of alternative splicing in the spatial relationship between *CD8* ^+^ *CXCL13* ^+^ Tex cells and *C1QC* ^+^ TAMs, we identified the spots that included these two cell types. Moreover, *CD74* exists in two major isoforms: *CD74*‐201 and *CD74*‐202. We grouped spatial spots into four groups: 1) High (*CD74*‐202 / *CD74*‐201 > 15), 2) Medium (1 < *CD74*‐202 / *CD74*‐201 ≦ 15), 3) Low (*CD74*‐202 / *CD74*‐201 ≦ 1), 3) Others (no *CD74*‐201 or no *CD74*‐202 and *CD74*‐201) (**Figure** **4**A,B,H,I). In the Before sample, there was no spot containing both cell types while there was no spot with a high *CD74*‐202/*CD74*‐201 ratio (Figure S11A–C, Supporting Information). However, in the After and AfterLN samples, the enrichment of *CD8* ^+^ *CXCL13* ^+^ Tex cells and *C1QC* ^+^ TAMs occurred (Figure 4C,D,J,K). Interestingly, the *C1QC* ^+^ TAMs occupied most of the spots in the AfterLN samples. Moreover, in both After and AfterLN samples, the *CD74*‐202/*CD74*‐201 ratio groups could significantly indicate the enrichment of *CD8* ^+^ *CXCL13* ^+^ Tex cells and *C1QC* ^+^ TAMs (Figure 4E,L). >73% and >85% of spots in high *CD74*‐202/*CD74*‐201 ratio group were enriched with *CD8* ^+^ *CXCL13* ^+^ Tex cells and *C1QC* ^+^ TAMs enrichment. Also, there was a significant difference in enrichment scores among all ratio groups (Figure 4F,M). In enrichment spots, the *CD74*‐202/*CD74*‐201 ratio was also significantly higher than in other spots (Figure 4G,N). Additionally, *CD8A*, *CXCL13*, *C1QC*, and *CD74* expression in the high, medium, and low *CD74*‐202/*CD74*‐201 ratio groups differed significantly (Figure S11D,G, Supporting Information). Spots with a high *CD74*‐202/*CD74*‐201 ratio didn't overlap with spots having only *C1QC* ^+^ TAMs or spots having only *CD8* ^+^ *CXCL13* ^+^ Tex cells (Figure S11E,F,H,I, Supporting Information).

To further validate the positive relationship between the *CD74*‐202/*CD74*‐201 ratio and the enrichment of *CD8* ^+^ *CXCL13* ^+^ Tex cells and *C1QC* ^+^ TAMs, we performed the same SiT sequencing and analysis pipeline on 13 tumor samples extracted from 13 ESCC patients. In the validation samples, 71.4% of spots in the high *CD74*‐202/*CD74*‐201 ratio group were enriched with *CD8* ^+^ *CXCL13* ^+^ Tex cells and *C1QC* ^+^ TAMs enrichment (Figure 4O). Similar to the After and AfterLN samples, a significant difference in enrichment score existed between the low *CD74*‐202/*CD74*‐201 ratio group and the medium *CD74*‐202/*CD74*‐201ratio group (Figure 4P). In all spatial spots of the 13 samples, the *CD74*‐202/*CD74*‐201 ratio of enrichment spots was significantly higher than in other spots (Figure 4Q). Therefore, we proposed that the occurrence of the elevated *CD74* isoform ratio that signifies the enrichment of *CD8* ^+^ *CXCL13* ^+^ Tex cells and *C1QC* ^+^ TAMs generally exists in the tumor and lymph node of ESCC patients.

Moreover, we performed BaseScope in situ hybridization for the *CD74*‐201 isoform and the *CD74*‐202 isoform (Figure 4R). Similar to the RNAscope results, the *CD74*‐202/*CD74*‐201 ratio in the LN significantly exceeded that in the enrichment fraction of the tumor. Additionally, the *CD74*‐202/*CD74*‐201 ratio in the LN was ≈30%, which is near the enrichment cell fraction of the *CD74*, *C1QC*, and *CXCL13* mRNAs (Figure 4S). However, while the *CD74*‐201 isoform was detected in both LN and tumor samples, the *CD74*‐202 isoform remained undetected in the tumor sample (Figure 4T).

To further explore the reason for the predictive power of the *CD74* isoform, we investigated the structural differences between the two isoforms. We examined the secondary structures of the two isoforms and predicted their 3D structures by using AlphaFold2 (Figure S12A,B, Supporting Information). Both isoforms had MHC class II‐associated invariant chain domains. The *CD74*‐201 isoform displayed an additional thyroglobulin type‐1 domain, leading to alterations in secondary structure and solvent accessibility (Figure S12C–F, Supporting Information). Our AlphaFold2‐predicted 3D structure revealed similarities in the shared amino acids, while the *CD74*‐201 isoform exhibited an additional beta‐strand and helix formed by extra amino acids. However, the *CD74*‐201 isoform had an additional thyroglobulin type‐1 domain, which changed the secondary structure and solvent accessibility (Figure S12C–F, Supporting Information). Our AlphaFold2‐predicted 3D structure revealed similarities in the shared amino acids, while the *CD74*‐201 isoform exhibited an additional beta‐strand and helix formed by extra amino acids.

In aggregate, high consistency occurred between the elevated *CD74* isoform ratio and the enrichment of *CD8^+^ CXCL13^+^* Tex cells and *C1QC* ^+^ TAMs after chemotherapy, attributable to structural differences in *CD74* isoforms. Notably, the *CD74*‐202/*CD74*‐201 ratio in LNs significantly surpassed that observed in the enrichment fraction within the tumor.

### 2.5 The *CD74* Isoform Ratio Exhibits a Positive Correlation with T‐cell Fate and Clonal Diversity

**Figure 5:**
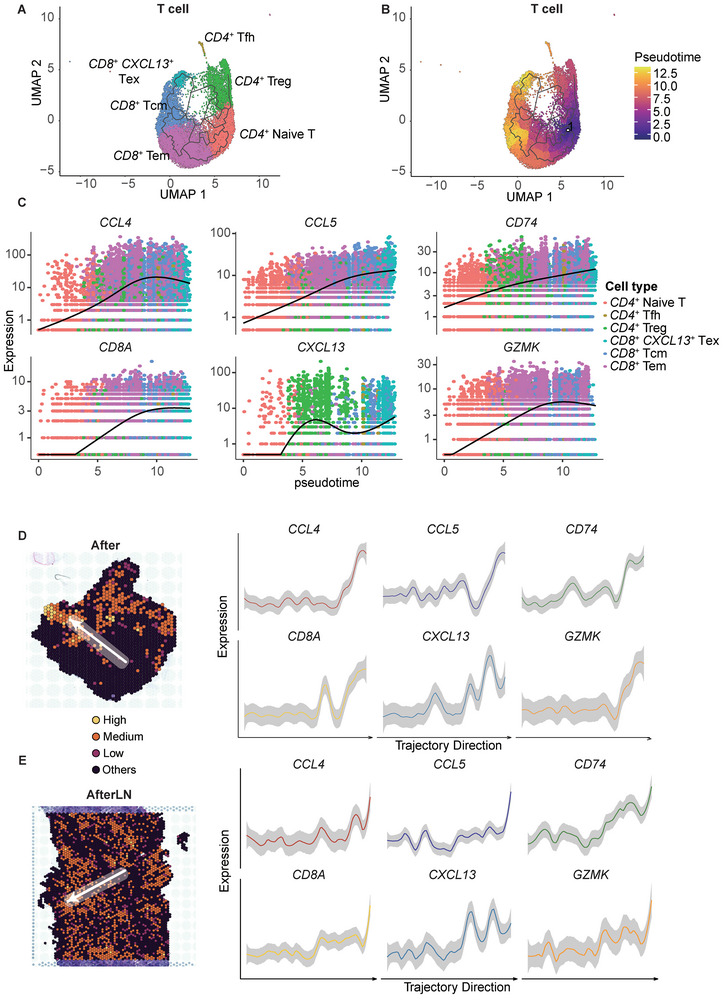
*CD74* expression is positively related to T‐cell fate and clonal diversity. A) UMAP of the cell type clustering of T cells in single‐cell transcriptomic spots. The colors indicate the cell type. B) Pseudotime plot of T cells in single‐cell transcriptomic spots. The colors indicate the pseudotime. C) Changes in *CCL4*, *CCL5*, *CD74*, *CD8A*, *CXCL13*, and *GZMK* expression levels with pseudotime. The colors indicate the cell types. D,E) Expression of *CCL4*, *CCL5*, *CD74*, *CD8A*, *CXCL13*, and *GZMK* according to the direction indicated by the arrow in the After and AfterLN groups. The arrows indicate a low‐to‐high ratio.

To verify the *CD8* ^+^ T‐cell trajectory, we performed single‐cell trajectory analysis and pseudotime reconstruction (**Figure** **5**A,B). Both *CD8* ^+^ *CXCL13* ^+^ Tex cells and *CD8* ^+^ Tem cells arose after *CD8* ^+^ Tcm cells. *CD8* ^+^ Tem cells exhibited different transition states. Additionally, along with the *CD8* ^+^ T‐cell trajectory, the expression levels of *CCL4, CCL5, CD8A*, and *GZMK* tended to increase, while *CXCL13* was expressed only in *CD8* ^+^ *CXCL13* ^+^ Tex cells and some *CD8* ^+^ Tcm cells (Figure 5C). To explore the relationship between clonal space homeostasis of T cells and *CD74* expression, we performed single‐cell RNA T‐cell receptor (TCR) sequencing. Information on TCR clonality and diversity was assigned to each cell. All cells were classified by the abundance of the corresponding clonotype (Figure S13A, Supporting Information). Among all the T cells, *CD8* ^+^ *CXCL13* ^+^ Tex cells were the only cell type with hyper‐expanded clonotype abundance. The abundance of the clonotype in most *CD4* ^+^ naïve T cells was low (rare), while the abundance of the clonotype in *CD4* ^+^ Tfh cells was high. Examination of the clonal overlap between each T‐cell subtype revealed that *CD8* ^+^ *CXCL13* Tex cells were strongly related to *CD8* ^+^ Tcm cells, while CD4^+^ Treg cells were strongly related to *CD4* ^+^ Tfh cells (Figure S13B–D, Supporting Information). The above results revealed that high exhaustion and low proliferation were positively related to high TCR diversity. Additionally, concomitant with the increase in TCR colonality and diversity, *CD74* expression increased. We validated that TCR signaling and *CD74* expression are positively related to T‐cell fate and are sufficient to promote the proliferation of T cells.

To further validate the relationship between the *CD74* isoform ratio and T‐cell fate, we analyzed the same changes in expression trends in the spatial expression data (Figure 5D,E). Transitioning from a low to a high ratio, there was a consistent elevation in the expression levels of *CCL4*, *CCL5*, *CD74*, *CD8*A, *CXCL13*, and *GZMK*. This expression pattern closely mirrored that of the *CD8* ^+^ *CXCL13* ^+^ Tex trajectory. Consequently, we proposed that the *CD74* isoform ratio is also positively related to TCR clonality and T‐cell fate.

### 2.6 *C1QC* ^+^ TAMs Play an Immunosuppressive Role through MIF‐CD74 + CD44 in Other Epithelial Cancers

**Figure 6:**
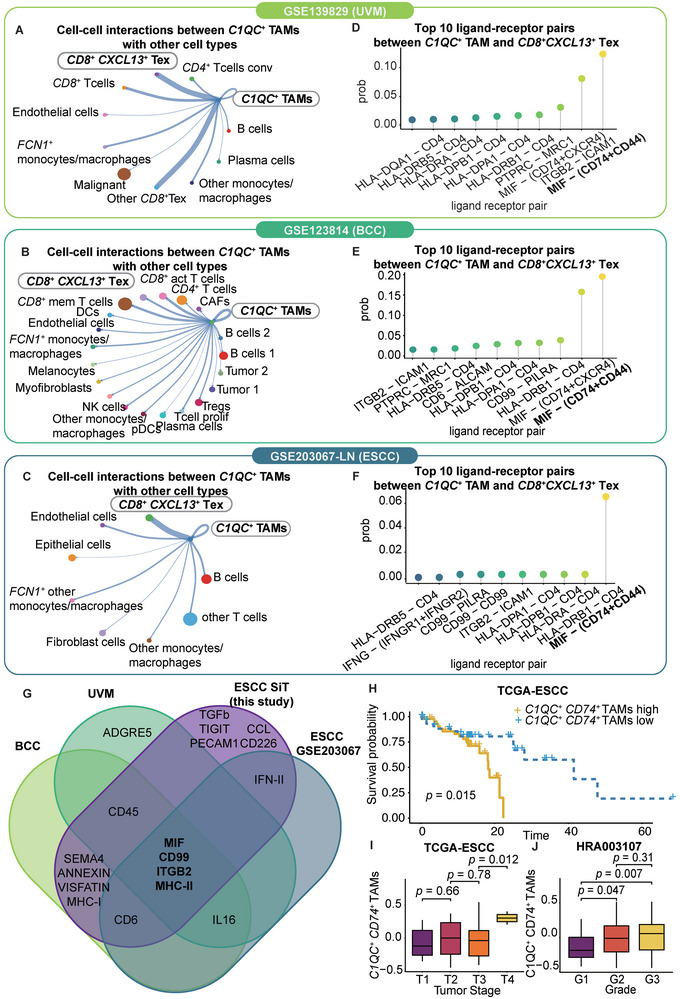
*C1QC* ^+^ TAMs play an immunosuppressive role through MIF‐CD74+CD44 in other epithelial cancers. A–C) Cell crosstalk diagram of all cell types in the GSE139829 dataset, GSE123814 dataset, and GSE203067 dataset. With respect to the GSE203067 dataset, only LN samples are shown. The width of the line indicates the strength of the interaction. The size of each cell type dot indicates the number of cells. The color of each cell type dot indicates the cell type. D–F) Lollipop plot of the top 10 ligand‐receptor pairs between *CD8^+^ CXCL13^+^* Tex cells and *C1QC^+^* TAMs in the GSE139829, GSE123814, and GSE203067 datasets. The colors indicate the ligand‐receptor pairs. G) Venn diagram of the top ligand‒receptor pairs between *CD8^+^ CXCL13^+^* Tex cells and *C1QC^+^* TAMs in our ESCC dataset, the UVM (GSE139829) dataset, the BCC (GSE123814) dataset and the ESCC (GSE203067) dataset. H) Kaplan–Meier curves for OS in the 90 patients in the TCGA‐ESCC cohort stratified according to high (*n =* 44) and low (*n =* 46) GSVA scores for the *C1QC^+^ CD74* ^+^ TAM marker. I) Boxplot of the GSVA score of the *C1QC^+^ CD74* ^+^ TAM marker in the 154 patients in the HRA003107 cohort for the T1 (*n =* 5), T2 (*n =* 77), T3 (*n =* 62) and T4 (*n =* 10) tumor stages. *P* value was calculated by an unpaired two‐tailed Student's t‐test. J) Boxplot of the GSVA scores of the *C1QC^+^ CD74* ^+^ TAM marker in the 155 patients in the HRA003107 cohort for grades G1 (*n =* 22), G2 (*n =* 84) and G3 (*n =* 49). *P* value was calculated by an unpaired two‐tailed Student's t‐test.

To validate the interaction between *C1QC* ^+^ TAMs and *FCN1* ^+^ monocytes/macrophages, we utilized three single‐cell datasets: 1) GSE139829, 2) GES12814 and 3) GSE203067. The GSE139829 and GSE12814 datasets were obtained from TISCH2.^[^ ^27^, ^28^, ^29^, ^30^ ^]^ For the GSE123814 dataset, uveal melanoma (UVM) samples from eleven patients were subjected to single‐cell sequencing.^[^ ^27^ ^]^ Despite a high metastasis rate, uveal melanoma was insensitive to immune checkpoint therapy. Three patients in this dataset had metastases, while eight patients had primary tumors only. The macrophages in the original annotation were clustered as *C1QC* ^+^ TAMs, *FCN1* ^+^ monocytes/macrophages, or other monocytes/macrophages (Figure S14A, Supporting Information). *C1QC* ^+^ TAMs represented ≈5% of all cells (Figure S15A, Supporting Information). Like those in ESCC, UVM TAMs could be clearly split by *C1QC* and *FCN1* expression, and *CD8* ^+^ *CXCL13* ^+^ Tex cells expressed *CXCL13* (Figure S14D,G, Supporting Information). Although there was no significant difference in the *C1QC* ^+^ TAM and *CD8* ^+^ *CXCL13* ^+^ ratio between primary patients and metastatic patients, the ratio of these two cell types exhibited the same trend in both groups and was greater in metastatic patients (Figures S14J,M, S15D,G, Supporting Information). However, *CD74* expression did not differ between primary patients and metastatic patients (Figure S14P, Supporting Information). Like in ESCC patients, UVM patients’ *C1QC* ^+^ TAMs interacted strongly with *CD8* ^+^ *CXCL13* ^+^ Tex cells (**Figure** **6**A).

For the GES12814 dataset, advanced basal cell carcinoma (BCC) samples from eleven patients were subjected to scRNA‐seq before and after anti‐PD‐1 therapy.^[^ ^28^ ^]^ We clustered the cells originally annotated as monocytes/macrophages into three types of TAMs: *C1QC* ^+^ TAMs, *FCN1* ^+^ monocytes/macrophages, and other monocytes/macrophages (Figure S14B, Supporting Information). *C1QC* and *FCN1* were found to be distinct markers for TAM clustering (Figure S14E, Supporting Information). *CXCL13* was highly expressed in *CD8* ^+^ *CXCL13* ^+^ Tex cells (Figure S14H, Supporting Information). *CD8* ^+^ T cells were the most abundant cells in all the samples (Figure S15B,E,H, Supporting Information). Furthermore, the *C1QC* ^+^ TAM ratio increased in the responsive patients before treatment and after treatment, and *CD8* ^+^ *CXCL13* ^+^ Tex cells exhibited the same trend (Figure S14K,N, Supporting Information). Remarkably, there were no *C1QC* ^+^ TAMs in the untreated tumors of responsive patients. Before treatment, nonresponsive patients had a significantly greater *C1QC* ^+^ TAM ratio than responsive patients. Moreover, *CD74* expression showed trends similar to those of *C1QC* ^+^ TAMs. *CD74* expression levels increased in responsive patients after treatment. Compared with responsive patients, nonresponsive patients had significantly greater *CD74* expression levels (Figure S14Q, Supporting Information). Furthermore, *C1QC* ^+^ TAMs from BCC patients interacted frequently with *CD8* ^+^ *CXCL13* ^+^ Tex cells (Figure 6B).

We analyzed twelve ESCC patients from the single‐cell RNA‐seq dataset GSE203067, which included four primary tumor samples, four adjacent normal tissue samples, three lymph nodes with metastasis samples, and one lymph node without metastasis sample.^[^ ^30^ ^]^ *CD8* ^+^ *CXCL13* ^+^ T cells accounted for 12% of the total cells in this cohort (Figure S15C, Supporting Information). We reclustered the cells originally annotated as monocytes/macrophages into three populations of TAMs: *C1QC* ^+^ TAMs, *FCN1* ^+^ monocytes/macrophages, and other monocytes/macrophages (Figure S14C,F, Supporting Information). *C1QC* and *FCN1* served as distinct markers for defining these TAM clusters (Figure S14F, Supporting Information). *C1QC* ^+^ TAMs were found at a higher ratio in the ESCC LNM cohort than in the other patient cohorts (Figure S15F–I, Supporting Information). *CXCL13* was highly expressed in *CD8* ^+^ *CXCL13* ^+^ Tex cells (Figure S14I, Supporting Information). Moreover, the ratio tended to be greater in lymph nodes harboring metastases than in those without metastases (Figure S14L, Supporting Information). Studies with larger sample sizes are warranted to validate whether *C1QC* ^+^ TAMs are truly enriched in lymph nodes during ESCC progression. Additionally, the levels of *CD8* ^+^ *CXCL13* ^+^ Tex cells and *CD74* expression in *C1QC* ^+^ TAMs showed similar trends (Figure S14O,R, Supporting Information). Critically, *C1QC* ^+^ TAMs from ESCC lymph nodes in this dataset interacted frequently with *CD8* ^+^ *CXCL13* ^+^ Tex cells (Figure 6C).

In all three scRNA‐seq datasets, MIF‐(CD74+CD44) ranked as the highest‐probability ligand–receptor interaction (Figure 6D–F). Four interactions recurred across datasets: *MIF, CD99, ITGB2*, and *MHC‐II* (Figure 6G). Analysis of the TCGA‐ESCC and HRA003107 cohorts showed that *C1QC* ^+^ TAM marker expression was negatively correlated with patient survival, as determined by the gene set variation analysis (GSVA) score (Figure 6H). *C1QC* ^+^ TAMs were also associated with a higher tumor grade and a later stage in both cohorts (*p* < 0.05) (Figure 6I,J).

Collectively, the analyses of the UVM, BCC, and ESCC datasets validated the interaction between *C1QC* ^+^ TAMs and *CD8* ^+^ *CXCL13* ^+^ Tex cells across epithelial cancers, despite their distinct origins. *CD74* expression in *C1QC* ^+^ TAMs and the *C1QC* ^+^ TAM ratio may reveal the responses to PD‐1 blockade and metastasis. The recurrent interactions identified present valuable opportunities for further mechanistic interrogation.

## 3 Conclusion

Chemotherapy is one of the standard treatments for ESCC. However, this treatment approach faces significant challenges, primarily related to drug resistance and the propensity for metastasis. Although one recent study explored the molecular mechanisms of chemoresistance through scRNA‐seq, the TME of LNs has seldom been studied.^[^ ^31^ ^]^ Moreover, the interplay between spatial heterogeneity, chemoresistance, and metastatic potential remains an intriguing puzzle.

In our research, we harnessed the power of SiT. Our study not only identified isoforms as predictors of clinical outcomes and metastasis, but it also revealed the diverse isoform spatial distributions. Nevertheless, the temporal order of alternative splicing may result in identical expression level changes for the same isoforms. A prior study highlighted how alternative splicing can modulate ribosomal composition, ultimately shaping the spatial phenotype of cancer cells.^[^ ^20^ ^]^ Additional evidence has proven the strong relationship between alternative splicing and patient survival.^[^ ^20^, ^32^ ^]^ This underscores the temporal heterogeneity inherent in isoform biogenesis, which contributes to the observed spatial heterogeneity in isoforms.

Strikingly, through SiT, our exploration unveiled a distinct distribution of *CD74* isoforms across various regions, each showcasing unique patterns of enrichment. The two *CD74* isoforms exhibited different distributions, and the ratio of the two isoforms was strongly related to the enrichment of *C1QC* ^+^ TAMs and *CD8* ^+^ *CXCL13* ^+^ Tex cells. Considering the enrichment of *C1QC* ^+^ TAMs and *CD8* ^+^ *CXCL13* ^+^ Tex cells, the role of alternative splicing was examined.

Previous studies have demonstrated that TAMs engage *CD8* ^+^ T cells and participate in spatiotemporal interactions, which may be enhanced under hypoxic conditions.^[^ ^9^ ^]^ TAMs play a pivotal role in the intricate landscape of cancer immunity. Notably, the suppressive effect of TAMs on *CD8* ^+^ T cells has not been definitively determined in a spatial context.^[^ ^21^ ^]^ High numbers of TAMs are associated with poor prognosis of a variety of solid tumor types.^[^ ^33^ ^]^ The immunosuppressive role of TAMs in cancer progression and dissemination has been widely reported in many studies.^[^ ^34^, ^35^ ^]^ In the context of LNs, a recent study revealed the emergence of immunosuppressive *APOC1* ^+^ *APOE* ^+^ macrophages characterized by *C1QA* expression.^[^ ^35^ ^]^ Consistent with the findings of a previous study, our study similarly observed a preference for exhausted T cells for *C1QC* ^+^ TAMs. Narrowing down from the antigen‐specific synaptic contacts described in a previous study, we indicated that the MIF‐CD74 ligand–receptor pair is the key player in the *C1QC* ^+^ TAMs – *CD8* ^+^ *CXCL13* ^+^ Tex cell axis.^[^ ^9^ ^]^ We showed that *C1QC* ^+^ TAMs predict a poor prognosis in ESCC patients and show high enrichment with *CD8* ^+^ *CXCL13* ^+^ Tex cells in LN and tumor samples after chemotherapy.

In contrast to other TAMs, *C1QC* ^+^ TAMs exhibited greater *CD74* expression. Elevated *CD74* expression has been associated with adverse prognosis, disease progression, and metastasis in multiple cancer types.^[^ ^36^, ^37^, ^38^ ^]^ Beyond its role in chaperoning MHCII, cell surface CD74 also functions as a receptor for *MIF*.^[^ ^39^ ^]^ Our study showed that *C1QC* ^+^ TAMs colocalized with *CD8* ^+^ *CXCL13* ^+^ Tex cells through MIF‐CD74. Furthermore, MIF‐CD74 was always the highest probability ligand‐receptor pair in the ESCC, BCC, and UVM cohorts. According to a previous report, blocking MIF‐CD74 signaling in macrophages can restore antitumor immune responses in patients with metastatic melanoma.^[^ ^40^ ^]^ Additionally, in a mouse xenograft model, inhibition of the MIF‐CD74 interaction significantly suppressed tumor growth.^[^ ^41^ ^]^ Therefore, MIF‐CD74 may play an important role in the spatial codependency between *C1QC* ^+^ TAMs and *CD8* ^+^ *CXCL13* ^+^ Tex cells, and this role is shared with several epithelial cancer types.

*CD74* isoforms were proven to be related to *MIF* and patient survival. Macrophage‐released endogenous CD74 inhibits melanoma cell growth and stimulates apoptosis under IFN‐γ stimulatory conditions by inhibiting the MIF/CD74/AKT survival pathway.^[^ ^39^ ^]^ Structural analysis using AlphaFold2 revealed that both isoforms possessed MHC class II‐associated invariant chain domains. However, the *CD74*‐201 isoform contains an additional thyroglobulin type‐1 domain, which was predicted to change the secondary structure and solvent accessibility. Hence, we hypothesized that *CD74*‐201 might confer an advantage in T‐cell recruitment based on its unique structure.

To validate our conclusions and extend their applicability, further analysis involving larger cohorts is imperative. Also, the pseudotime analysis R package was used for inferring cellular dynamic trajectories and quantifying the cell fate in this research. The experimental constraints need to be addressed for assessing the gene expression along with the cell fate. In addition, although isoform quantitative analysis identified the key *CD74* isoform, the general alternative splicing mechanism at the spatial level has not been determined. To unravel these underlying regulatory processes, we advocate for the utilization of additional R packages or bioinformatic tools specifically tailored for spatial isoform information. Additionally, SiT still has shortcomings, such as low UMI labeling and low numbers of barcoded reads, which represent only 60–70% of the total reads. However, further spatial isoform sequencing technology with increased accuracy is needed. *CD74* is also expressed in other cell types, including epithelial cells, within lymph node metastases of ESCC.^[^ ^37^ ^]^ Therefore, further exploration is needed to gain deeper insights into the potential regulatory mechanisms of *CD74* in shaping the lymph node tumor microenvironment during metastasis. Elucidating the functions of *CD74* in different immune and epithelial cells could provide new insight into its role in coordinating the metastatic niche.

In addition, our findings may contribute to the development of personalized metastasis diagnosis, mRNA drug therapy, and mRNA‐based molecular imaging. Antisense oligonucleotides (ASOs) are small, synthetic, single‐stranded nucleic acid polymers that offer the ability to selectively target specific isoforms. ASO drugs possess the unique advantage of targeting virtually any genetic component, even those traditionally deemed intractable or resistant to drug intervention, including small molecules and antibodies. Additionally, ASOs have other benefits, such as rapid production, long‐term effects, usefulness for rare diseases, and no risk of genotoxicity.^[^ ^42^ ^]^ Moreover, ASOs play an important role in molecular imaging. As an emerging technique, molecular imaging allows in situ visualization, categorization, and exploration of molecular biological processes within living organisms using modalities such as positron emission tomography (PET), magnetic resonance imaging (MRI), or computed tomography (CT).^[^ ^43^, ^44^ ^]^ Specific oligonucleotide imaging can be leveraged for therapy monitoring and evaluating treatment effectiveness.^[^ ^45^ ^]^

## 4 Experimental Section

### Experimental Design

To investigate the spatial isoform distribution in ESCC patients after chemotherapy, systematic research was conducted. ScRNA‐seq, TCR, and SiT (spatial isoform transcriptome) sequencing were performed on tumor tissues and LN tissues from one patient with metastasis and chemoresistance. The analysis of single‐cell and spatial transcriptome profiles focused on three main aspects: technique accessibility and data quality of SiT for solid tumors; single‐cell transcriptomic profiling (cell clustering, cell‐cell interaction analysis, TCR profiling, and pseudotime analysis); and spatial transcriptomic profiling (spatial gene expression pattern, spatial cluster interaction analysis, pseudotime analysis, spatial isoform classification, isoform distribution pattern, and isoform spatial relationship analysis). Validation was performed in four parts: SiT validation data from 13 tumor samples from 13 patients; RNAscope and BaseScope in situ validation in patients’ tissues; scRNA public database validation based on thirty‐four patients with three epithelial cancer types; and bulkRNA public database validation based on 245 patients with ESCC. This study was reviewed and approved by the relevant ethics committees, including the Institutional Review Boards of Shanxi Medical University and the independent ethics committee of the National Cancer Center, Cancer Hospital, Chinese Academy of Medical Sciences (CHCAMS) (2016LL106, ChiCTR2000040034). Written informed consent was obtained from each participant.

### 10X Single‐cell RNA Sequencing and TCR

scRNA‐seq was performed by using the 10X Chromium Single‐cell 5′ v2 Kit (10X Genomics, Pleasanton, CA) following the manufacturer's protocol. Sequencing libraries were prepared according to the manufacturer's protocol. Sequencing was performed on a HiSeq 4000 platform (Illumina, Inc., San Diego, CA). The raw sequencing data were processed with the CellRanger pipeline (version 7.0.0, 10X Genomics) and mapped to the hg19 reference genome to generate matrices of gene counts by cell barcodes. Additionally, the TCR library was generated from single‐cell V(D)J sequencing. The raw single‐cell V(D)J sequencing data were processed with the CellRanger pipeline (version 7.0.0, 10X Genomics) and mapped to vdj_GRCh38_alts_ensembl‐7.0.0.

### 10X Genomics Visium Spatial Library Construction

We followed the SiT pipeline with some modifications. Hematoxylin and eosin (H&E) staining of tumor sections was performed first. A Visium Spatial Tissue Optimization Slide & Reagent Kit (10X Genomics, Pleasanton, CA, USA) was used to optimize permeabilization conditions for tumor and LN tissue samples. Spatially barcoded full‐length cDNA was generated using a Visium Spatial Gene Expression Slide & Reagent Kit (10X Genomics) following the manufacturer's protocol. The cDNA amplification temperatures, times, and cycles used were listed in Tables S2 and S3 (Supporting Information). A fraction of each cDNA library was subjected to nanopore sequencing, whereas 10 µL was subjected to fragmentation, adapter ligation, and indexing according to the 10X Genomics Visium library preparation protocol.

### Oxford Nanopore Sequencing and Illumina Sequencing

cDNA was amplified through PCR with KAPA HiFi HotStart ReadyMix (KK2602, Merck) for 10 cycles following the kit instructions. The samples were then cleaned with Beckman Coulter SpriSelect Beads (Cat# B23318) at a 0.6x ratio, after which the cDNA was eluted in 50 µL of nuclease‐free water. The size distribution was checked using an Agilent Fragment Analyzer Large Fragment Kit (Cat# DNF‐464‐0500). An Oxford Nanopore‐compatible library was produced using 500 ng of cDNA derived from 10x Genomics Visium following the Genomic DNA by Ligation protocol (SQK‐LSK109) from Oxford Nanopore with the following modifications. End repair was carried out by omitting NEBNext FFPE DNA Repair, and the incubation times were extended to 10 min at 20 °C and 10 min at 65 °C. The loading input for PromethION was increased to 150 fmol, sequencing was carried out for 20 h, and base calling was performed using Guppy (version 3.2.10).

### Illumina Sequencing Data Processing

For short‐read sequencing, the sequencing data were processed using SpaceRanger software (version 1.1.0) with default parameters and mapped to the human genome (hg38). Gene expression was quantified based on the unique molecular identifier (UMI). For quality control, low‐quality spots whose gene count was <1000 or whose mitochondrial gene ratio was >5% were removed.

### Oxford Nanopore Data Processing

Nanopore reads were processed according to the SiT protocol, which refers to the scNaUmi‐seq protocol, with slight modifications.^[^ ^46^ ^]^ All reads were scanned for poly(A/T) tails and 3′ adapter sequences to determine the orientation of the reads and strand specificity. The scanned reads were subsequently aligned to the human genome (hg38) with minimap2 (version 2.17) in spliced alignment mode.^[^ ^47^ ^]^ Spatial barcodes and UMIs were subsequently assigned to nanopore reads using the strategy and software previously described for single‐cell libraries. The consensus sequence per molecule (UMI) was computed according to the number of available reads for the UMI using the ComputeConsensus sicelore‐2.0 pipeline (https://github.com/ucagenomix/sicelore). Consensus cDNA sequences were aligned to the human genome (hg38) built with minimap2 (version 2.17) in spliced alignment mode. SAM records matching known genes were analyzed for matching Gencode vM24 transcript isoforms (same exon makeup). To assign a UMI to a Gencode transcript, a full match was required between the UMI and the Gencode transcript exon‐exon junction layout authorizing a two‐base margin of added or lacking sequences at exon boundaries to allow for indels at exon junctions and imprecise mapping by minimap2.

### Spatial Multiassay Storage

Raw gene expression matrices generated by Space Ranger were processed using R (version 4.1.0) and the Seurat package (version 4.2.1).^[^ ^48^ ^]^ We created Seurat objects for each sample with different assays for the analysis as follows: i) “Spatial” containing gene‐level raw short‐read data from the Space Ranger output, ii) “ISOG” containing the gene‐level Nanopore long‐read data, and iii) “ISO” containing isoform‐level transcript information where only the molecules where all exons were observed were kept.

### Differential Splicing Detection and Isoform Classification

The Seurat FindMarkers function (logfc.threshold = 0.25, test.use = “Wilcox”, min.pct = 0.1) was used to detect genes showing at least two isoforms as markers of different brain regions via the nanopore isoform‐level “ISO” assay. The results were filtered for nonmajority isoforms, i.e., not the isoform showing the highest bulk expression, requiring a Bonferroni‐adjusted P value of ≤0.05. We classified the isoforms into four types based on the relationship between the isoform and the corresponding gene and spatial distribution pattern: 1) multi all: isoforms with various “alternate isoforms” for a corresponding gene that were distributed to all spatial niches; 2) multi one: isoforms with various “alternate isoforms” for a corresponding gene that were uniquely expressed in one spatial niche; 3) multi others: isoforms with various “alternate isoforms” for a corresponding gene that were distributed to two or more spatial niches but not all spatial niches; and 4) single: an isoform that was the only isoform of a corresponding gene.

### 3D Isoform Expression Patterns

To observe all the isoforms of RPS9 in the same 3D plot, the expression data for the three isoforms from the Seurat object was first extracted and combined into one assay. Then, the tissue sections were masked and aligned into the Seurat object by using the Create3DStack function to create the 3D stack from the aligned images. The FeaturePlot3D function of the STutility package was used for visualizing the stacked isoforms in 3D.^[^ ^49^ ^]^

### Transcriptome Correlation

We computed and minimized the physical distance between spots to define the pair of spots showing the smallest distance between sections. We then computed the whole‐transcriptome correlation per pair of spots using the cor.test function of the Stats R package with gene‐level short‐read (spatial assay) and long‐read (ISOG assay) UMI count matrices.

### Single‐cell RNA‐seq Data Processing

After alignment and quantification through CellRanger, the following quality control procedure was applied. Cells with <500 UMIs and above 4000 UMIs were filtered out. Additionally, cells with more than 5% mitochondrial gene expression were filtered. Doublets were identified and filtered using the DoubletFinder package.^[^ ^50^ ^]^ Batch effects derived from different samples were adjusted using the Harmony package.^[^ ^51^ ^]^

### Dimension Reduction and Unsupervised Clustering

By using the NormalizeData, FindVariableFeatures, and ScaleData functions, the counts were normalized. Dimension reduction was performed by the RunPCA function. For visualization, the dimensionality was further reduced by using the runUMAP function.

### Contact Frequency Calculation

The six closest spatial spots to any given spatial spot were defined as their spatial spot neighbors. Spatial niches were clustered through previous unsupervised clustering. Each spot was labeled with its spatial niche. For each spatial spot, contact frequency was defined as the number of different spatial niches in its spatial spot neighbors. Therefore, the number of neighboring spatial niches were counted that differed from their own spatial niche for each spot. This frequency was computed for each spatial spot across all samples. The boxplot was generated to compare the contact frequency of spatial spots in each sample by ggplot2 R package.^[^ ^52^ ^]^

### Cell Type Annotation

All the clusters were annotated by known marker genes (*COL1A1*, *COL1A2*, *CD19*, *MS4A1*, *CD79B*, *CD79A*, *CST3*, *LYZ*, *HPGDS*, *MS4A2*, *CD3E* and *MZB1*). In monocytes/macrophages, clusters were annotated based on previously published markers (*FCN1*, *S100A9*, *S100A8*, *FCGR3A*, *LST1*, *LILRB2*, *IBHBA*, *IL1RN*, *CCL4*, *NLRP3*, *EREG* and *IL1B* for *FCN1* ^+^ monocytes/macrophages; *LYVE1*, *PLTP*, *C1QC*, *C1QB* and *C1QA* for *C1QC* ^+^ TAMs).

### Differential Gene Expression Analysis and Single‐cell TCR Analysis

All differential gene expression analyses and marker gene identification were performed with the Seurat R package. The differentially expressed genes for each cluster compared with those for all other cells were identified using the FindAllMarkers function. We annotated the Seurat object with clonal abundance with the scRepertoire R package.^[^ ^53^ ^]^ A volcano plot was generated using the EnhancedVolcano package.^[^ ^54^ ^]^

### Gene Ontology (GO) Enrichment Analysis

GO enrichment analysis was performed using the clusterProfiler (version 4.2.2) R package, and a Benjamini–Hochberg‐adjusted *p <* 0.01 was considered to indicate statistical significance. We used the dot plot function to visualize the enrichment results.^[^ ^55^ ^]^

### Gene Set Variation Analysis

The hallmark pathway gene sets were exported from the MSigDB database. The GSVA score was calculated based on the top 50 *C1QC^+^* TAM marker gene signatures in the TCGA‐ESCC cohort. Group classification was based on the mean GSVA score. The GSVA score was calculated by the GSVA package.^[^ ^56^ ^]^ For T cells, the gene set scores were calculated using the AddModuleScore function of the Seurat package. The module genes used were *GZMA*, *GZMA*, *GZMH*, *GZMM*, *GZMK*, *GNLY*, *PRF1* and *CTSW* (for cytotoxicity), *KIR2DL1*, *KIR2DL3*, *KIR3DL1*, *KIR3DL2*, *LILRB1* and *LAG3* (for HLA‐dependent inhibitory receptor); *PDCD1*, *SIGLEC7*, *CD300A*, *CD96*, *TIGIT* and *HAVCR2* (for HLA‐independent inhibitory receptor); *KIR2DL4*, *CD160* and *KLRC2* (for HLA‐dependent activating receptor); and *NCR3*, *NCR1*, *KLRK1*, *CRTAM* and *FCGR3A* (for HLA‐independent activating receptor).

### Single‐cell Trajectory Analysis and Cell–Cell Interaction Analysis

We used human ESCC single‐cell sequencing data from Zhang, Xiannian, et al. as a reference dataset.^[^ ^23^ ^]^ We filtered and retained relevant marker genes of the reference dataset with an AUC > 0.8. For each dataset, 100 cells were randomly sampled from all major cell types as input for the subsequent deconvolution analysis. For each sample, spatial spots were deconvoluted using SPOTlight (version 0.99.11).^[^ ^57^ ^]^

### CNV Estimation

We applied the InferCNV R package (version 1.10.1) using a moving average of 100 analyzed genes to estimate CNVs in each spot and at each analyzed gene/chromosomal location.^[^ ^58^ ^]^ For each sample, the niche with the highest CNV level was used as an independent reference.

### Spatial Trajectory Analysis and Niche‐Niche Interaction Analysis

All ligand‒receptor interaction analyses and spatial trajectory analyses were performed with stlearn. We imported the clustering and annotation data for all the samples from Seurat. We ranked the top 50 interacting pairs with the strongest interaction. We constructed a trajectory tree of niches that included *CD8* ^+^ *CXCL13* ^+^ Tex cells and *C1QC* ^+^ TAMs. By manually indicating a “pseudo” trajectory from a low *CD74* isoform ratio to a high *CD74* isoform ratio, a gene expression change pattern was constructed by using SPATA2.^[^ ^59^ ^]^

### Enrichment of CD8^+^ CXCL13^+^ Tex Cells and C1QC^+^ TAMs Identification and Enrichment Score Calculation

*CD8* ^+^ *CXCL13* ^+^ Tex cells and *C1QC* ^+^ TAMs were located by filtering the spots with marker expression > 0 (*CD8A*, *CXCL13*, *CD3E*, *PDCD1* for *CD8^+^ CXCL13^+^* Tex, *C1QC*, *LYZ*, *CD74*, *APOE*, *CSF1R* for *C1QC^+^* TAM). The enrichment was defined as spots and its nearest 6 spots contained *CD8^+^ CXCL13^+^* Tex cells and *C1QC^+^* TAMs. The enrichment score was calculated as the number of spots in the nearest six spots containing different cell types. If the spots containing both *CD8^+^ CXCL13^+^* Tex cells and *C1QC^+^* TAMs, The enrichment score represents the sum of scores calculated separately for *CD8^+^ CXCL13^+^* Tex cells and *CD8^+^ CXCL13^+^* Tex cells *C1QC^+^* TAMs interactions.

### Isoform Characterization and Protein Structure Prediction

The isoform domain annotations of the *CD74* isoforms were obtained from Pfam.^[^ ^60^ ^]^ The secondary structure and solvent accessibility annotation were obtained from PredictProtein.^[^ ^61^ ^]^ The protein structures of the *CD74* isoforms were predicted from the AlphaFold2 Colab notebook.^[^ ^62^ ^]^

### Survival Analysis and Clinical Information Correlation Analysis

RNA‐seq and clinical data were obtained from HRA003107 (WGS & RNA‐seq, https://ngdc.cncb.ac.cn/gsa‐human/browse/HRA003107) and TCGA‐ESCC. The survival curve was plotted using the survminer (version 0.4.9) R package.^[^ ^63^ ^]^ The GSVA score was calculated based on the top 50 *C1QC* ^+^ TAM marker gene signatures in the TCGA‐ESCC cohort. Group classification was based on the mean GSVA score. The GSVA score was calculated with the GSVA package.^[^ ^56^ ^]^

### Detection of Colocalized Cells by RNA In Situ Hybridization

RNAscope analysis of tissues was performed using an RNAscope Reagent Kit (Advanced Cell Diagnostics, Hayward, CA, USA). The RNA integrity of each sample was quality controlled with an RNAscope probe specific for *C1QC*, *CXCL13*, and *CD74* RNA. The samples were counterstained with DAPI. Representative images were digitally obtained using Vectra Polaris Fluorescence Whole Slide Scanning (PerkinElmer, Shelton, CT, USA). Tissue imaging analysis was performed by inForm (PerkinElmer, Shelton, CT, USA). Group comparisons of two isoforms between tumor and LN samples were performed using one‐way and two‐way analyses of variance (ANOVAs) with repeated measures comparisons when needed.

### Detection of the CD74 Isoform by BaseScope

The BaseScope Duplex assay from Advanced Cell Diagnostics was used to detect the specific *CD74* mRNA by in situ hybridization. Hydrogen peroxide, Protease IV treatment, and RNA in situ hybridization were performed using the BaseScope Duplex reagent kit (Advanced Cell Diagnostics, Newark, CA, USA). Specific *CD74*‐201 and *CD74*‐202 BaseScope probes were designed. The samples were counterstained with hematoxylin Gills I (GHS132‐1 L; Sigma) diluted to 50% in water (30‐second staining) and ammonium hydroxide 28–30 WT% (205840025; Acros Organics, Geel, Belgium) diluted to 0.02% in water (30‐second staining). Images were obtained using an Aperio AT2 scanner (Leica; zoom ×40) and analyzed with ImageScope software (Leica). The positive Pixel Count v9 program of ImageScope software was used for quantitative analysis of the relative area covered by each signal. Group comparisons of two isoforms between tumor and LN samples were performed using one‐way and two‐way ANOVAs with repeated measures comparisons when needed.

### Statistical Analysis

All statistical analyses were performed in R. Pre‐processing of data was described in the previous method section. Numerical results were reported as the means ± SEMs. A t‐test was used to assess the statistical significance of differences between means. *p <* 0.05 was defined as the threshold for statistical significance. The Benjamini–Hochberg (BH) method was used for P value correction in multiple tests. Detailed statistical methods in this paper can be found above.

## Conflict of Interest

The authors declare no conflict of interest.

## Author Contributions

The project was conceptualized by Y.Y., Y.H.Z., and Z.H.L. Methodology was developed by Y.Y., Y.H.Z., and Y.H.W. The investigation was conducted by Y.Y., Y.H.W., Y.L., X.Y., and Y.F.W. Visualization was handled by Y.Y., while supervision was provided by Y.H.Z., Y.F.W., and Z.H.L. Y.Y. was responsible for writing the original draft, and the review and editing were carried out by Y.Y., Y.H.Z., and Z.H.L.

## Supporting information

Supporting Information

Supporting Information

Supporting Information

## Data Availability

The data that support the findings of this study are openly available in the Genome Sequence Archive (GSA) at http://bigd.big.ac.cn/gsa, BioProject number PRJCA023185.
