# Supplementary material for: Spatial Isoforms Reveal the Mechanisms of Metastasis
Source: Adv Sci (Weinh). 2024 Sep 23;11(43):2402242. doi: 10.1002/advs.202402242 (PMC11578385; doi:10.1002/advs.202402242)
Supplement: Supplementary file 1 — Supporting Information [file ADVS-11-2402242-s002.pdf]

ADVANCED SCIENCE
Open Access

Supporting Information

for *Adv. Sci.*, DOI 10.1002/advs.202402242

Spatial Isoforms Reveal the Mechanisms of Metastasis

Yin Yin, Yuhao Wang, Xiao Yu, Yang Li, Yahui Zhao, Yanfeng Wang* and Zhihua Liu**

Supporting Information

Spatial Isoforms Reveal the Mechanisms of Metastasis

Yin Yin[1], Yuhao Wang[1], Xiao Yu[1], Yang Li[1], Yahui Zhao[1], Yanfeng Wang[2]* and Zhihua Liu[1]**

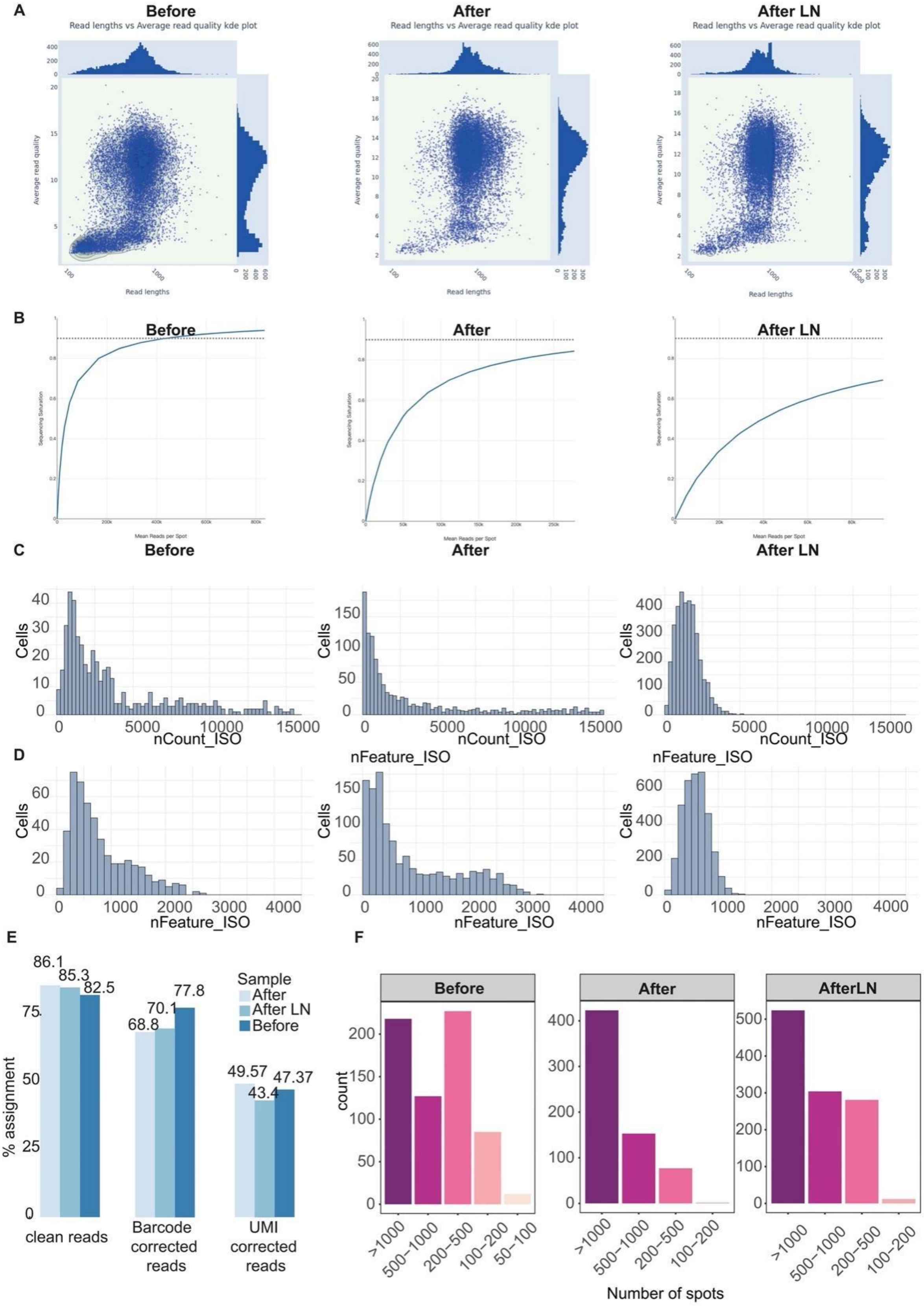

Figure S1. Overall quality of the SiT data for all samples.
(**A**) Read length and average read quality in all three samples. (**B**) Nanopore sequencing saturation curves showing the number of UMIs observed as a function of the number of Nanopore reads in all samples. (**C**) Number of cells with different numbers of mRNAs in all

samples. (**D**) Number of cells with different numbers of isoforms in all samples. (**E**) Percentage of assignment at each step of the workflow in all samples. Clean reads are expressed as a percentage of total reads; Barcode-corrected reads are expressed as a percentage of clean found reads with barcode correction; UMI-corrected reads are expressed as a percentage of barcode-corrected reads with UMIs. (**F**) Read counts with different numbers of spots in the three samples.

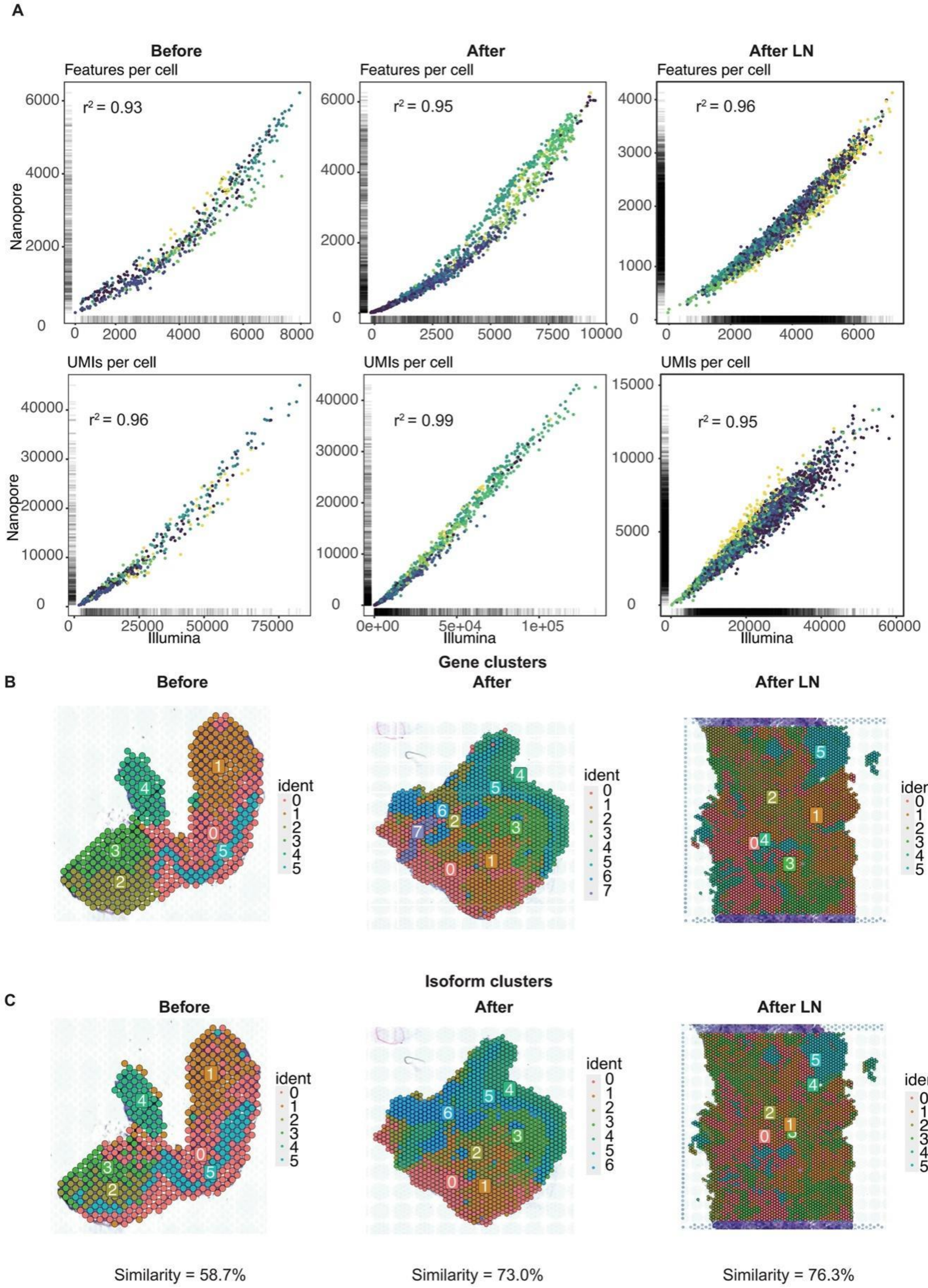

Figure S2. Expression and clustering relationship between short-read sequencing and long-read sequencing data.

(**A**) Correlations of UMIs per cell and feature per cell between short-read sequencing and long-read sequencing data. (**B**) Gene expression spatial clustering of all samples. The colors indicate the clusters. (**C**) Isoform expression spatial clustering of all samples. The colors indicate the clusters.

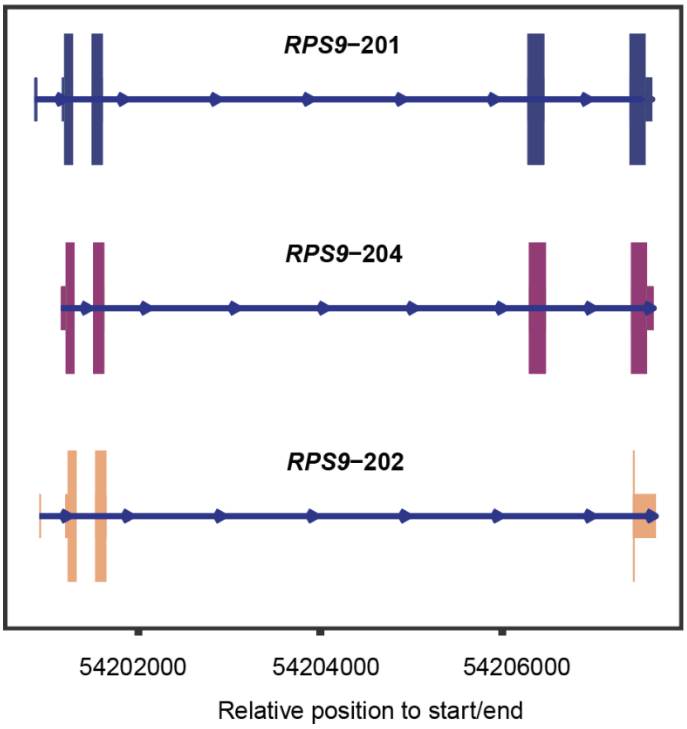

Figure S3. Structure and location of *RPS9* isoforms.

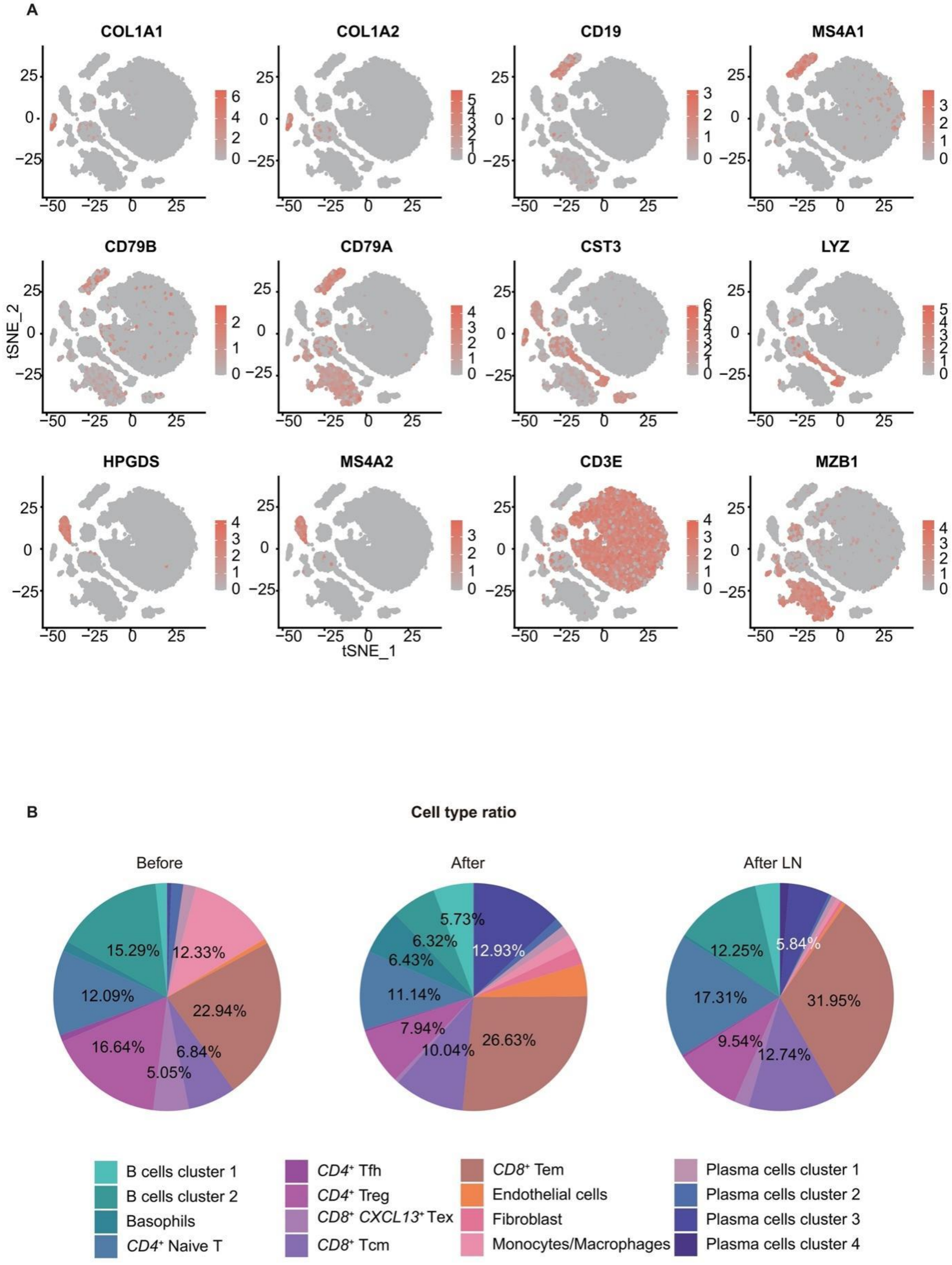

Figure S4. Clustering marker expression and cell type ratio in single-cell transcriptomics.

(**A**) TSNE plot of the gene expression data from single-cell transcriptomics. The colors indicate the expression level. The labels indicate the gene markers used for clustering. (**B**) Pie charts showing the cell type ratio. Ratio labels of <5% are omitted.

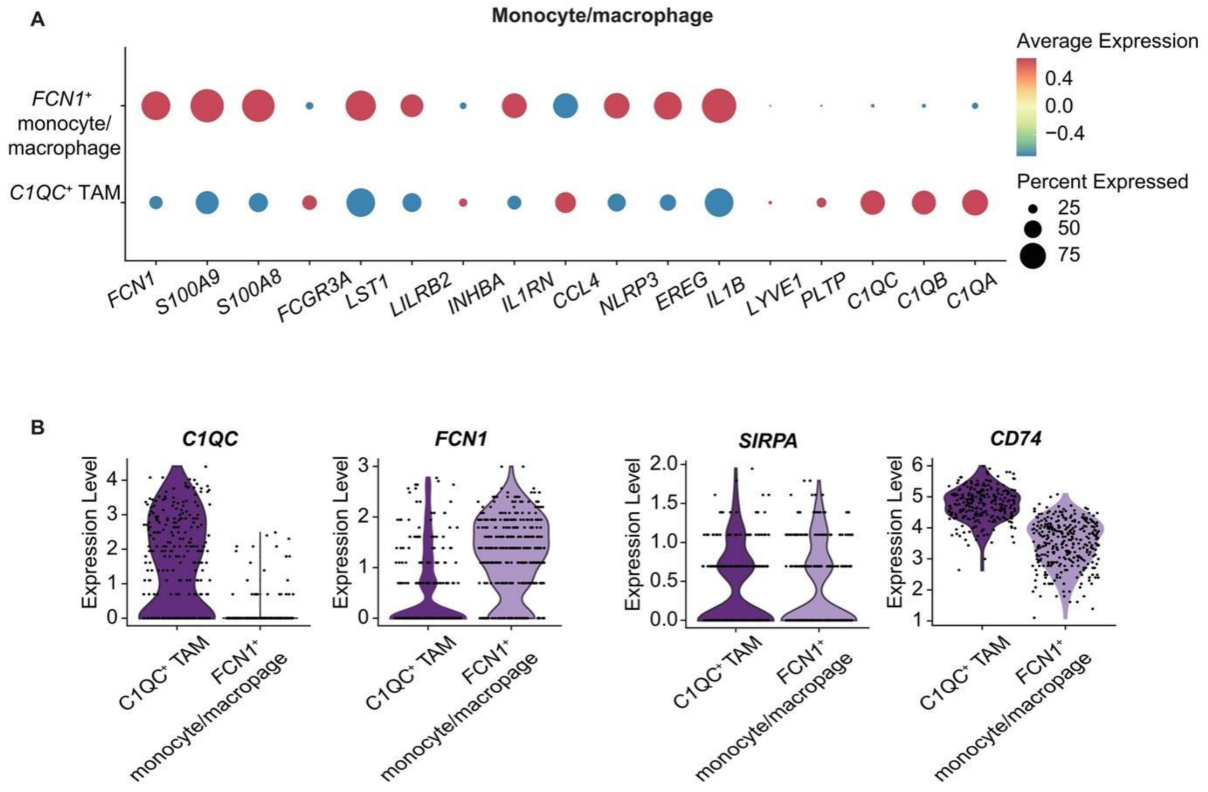

Figure S5. Clustering marker expression in monocytes/macrophages.
(**A**) Dot plot of marker expression in each monocyte/macrophage type. The dot size indicates the percentage of all cells with expression. The colors indicate the average expression level. (**B**) *FCN1*, *C1QC*, *CD74* and *SIRPA* gene expression in TAMs after normalization. The colors indicate the expression level.

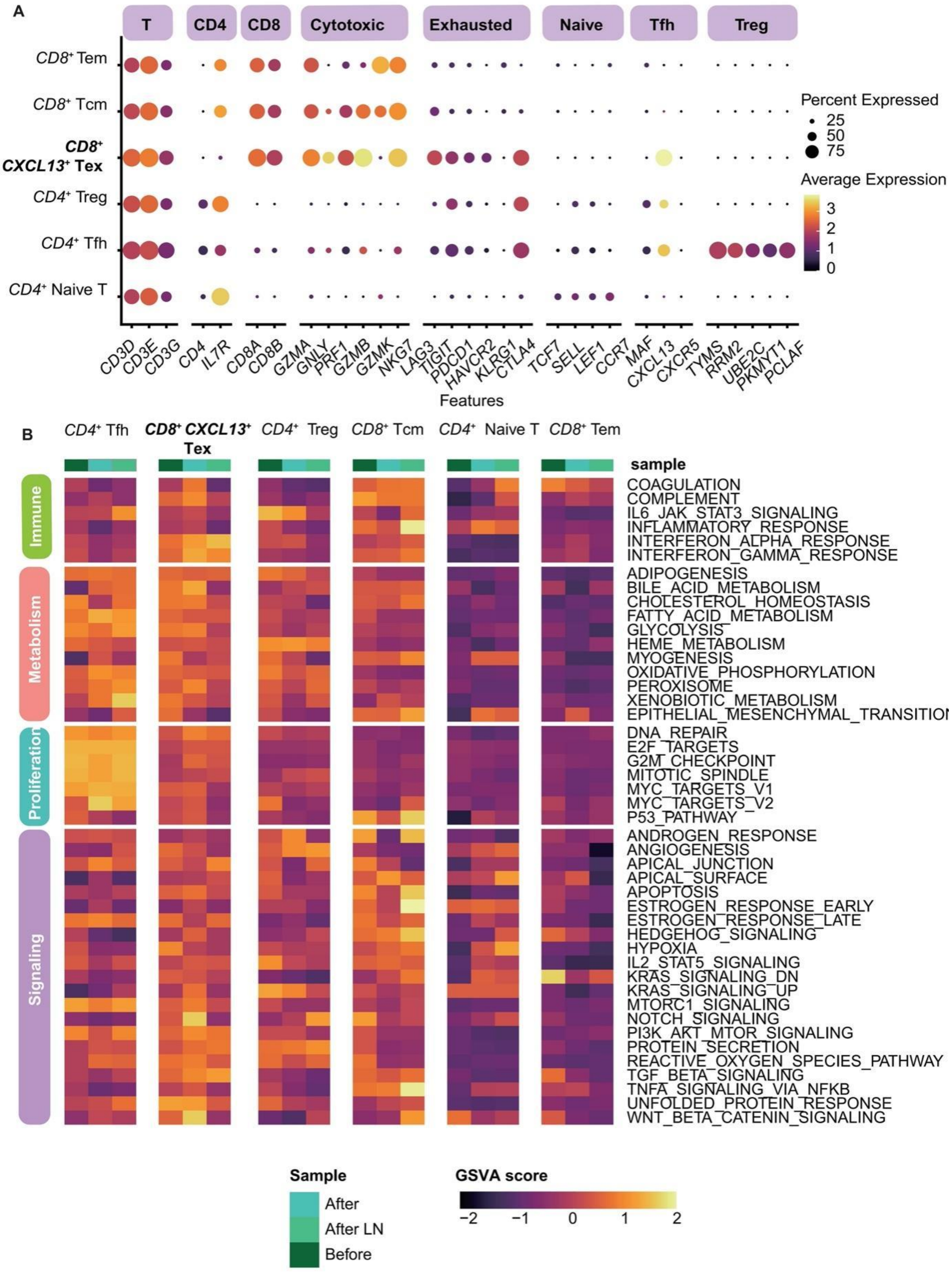

Figure S6. Clustering marker expression and hallmark pathways in T cells.
(**A**) Dot plot of marker expression in each cell type of T cells. The dot size indicates the percentage of all cells with expression. The colors indicate the average expression level. (**B**)

Heatmap of important tumor regulatory pathways in subclusters of T cells. The colors of the heatmap indicate the GSVA score.

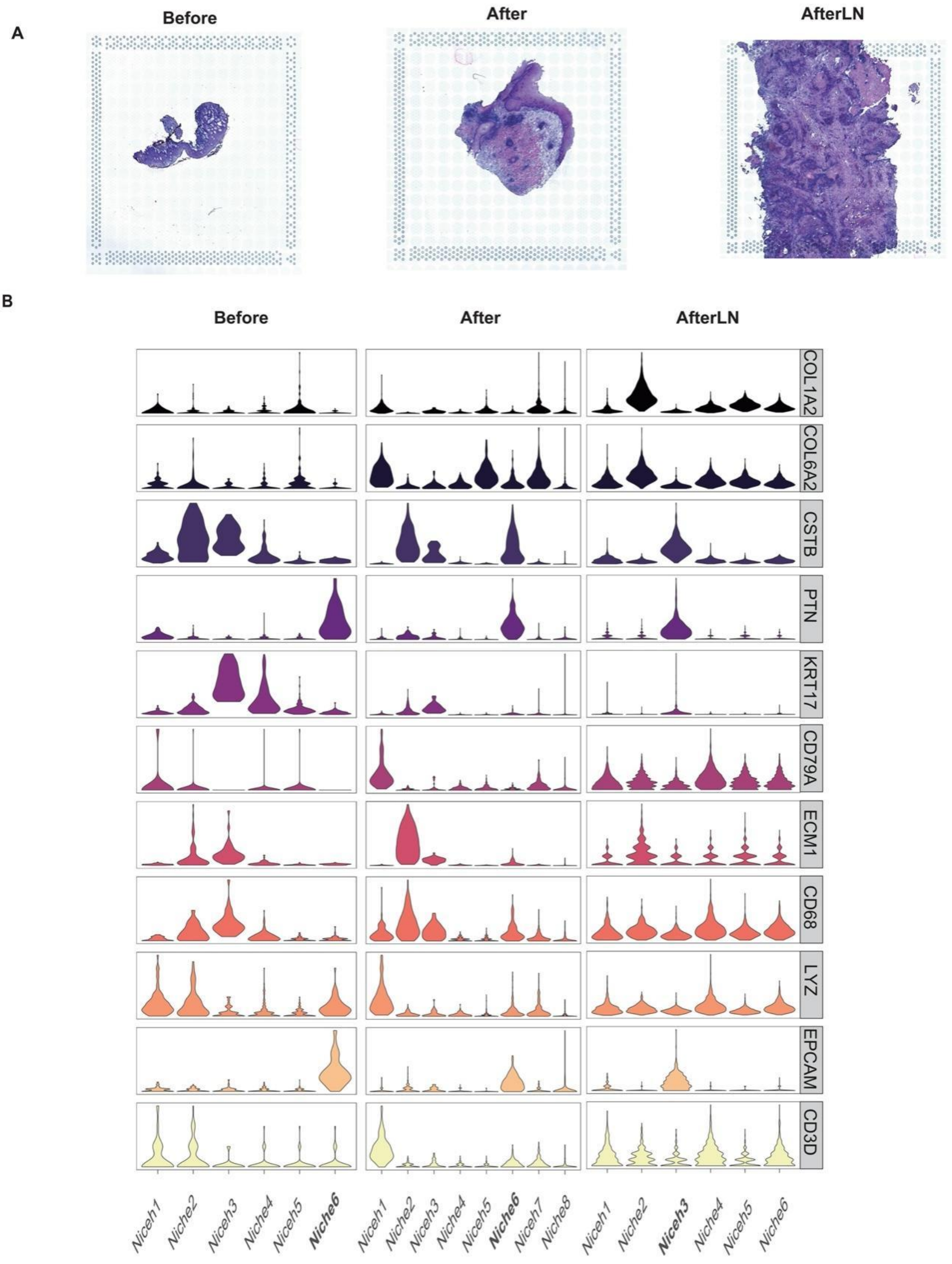

Figure S7. Spatial transcriptional characteristics.

(**A**) HE staining of tumor samples before treatment and after treatment and of the lymph nodes after treatment. (**B**) Single-cell cluster signature gene expression in the spatial transcriptomic data.

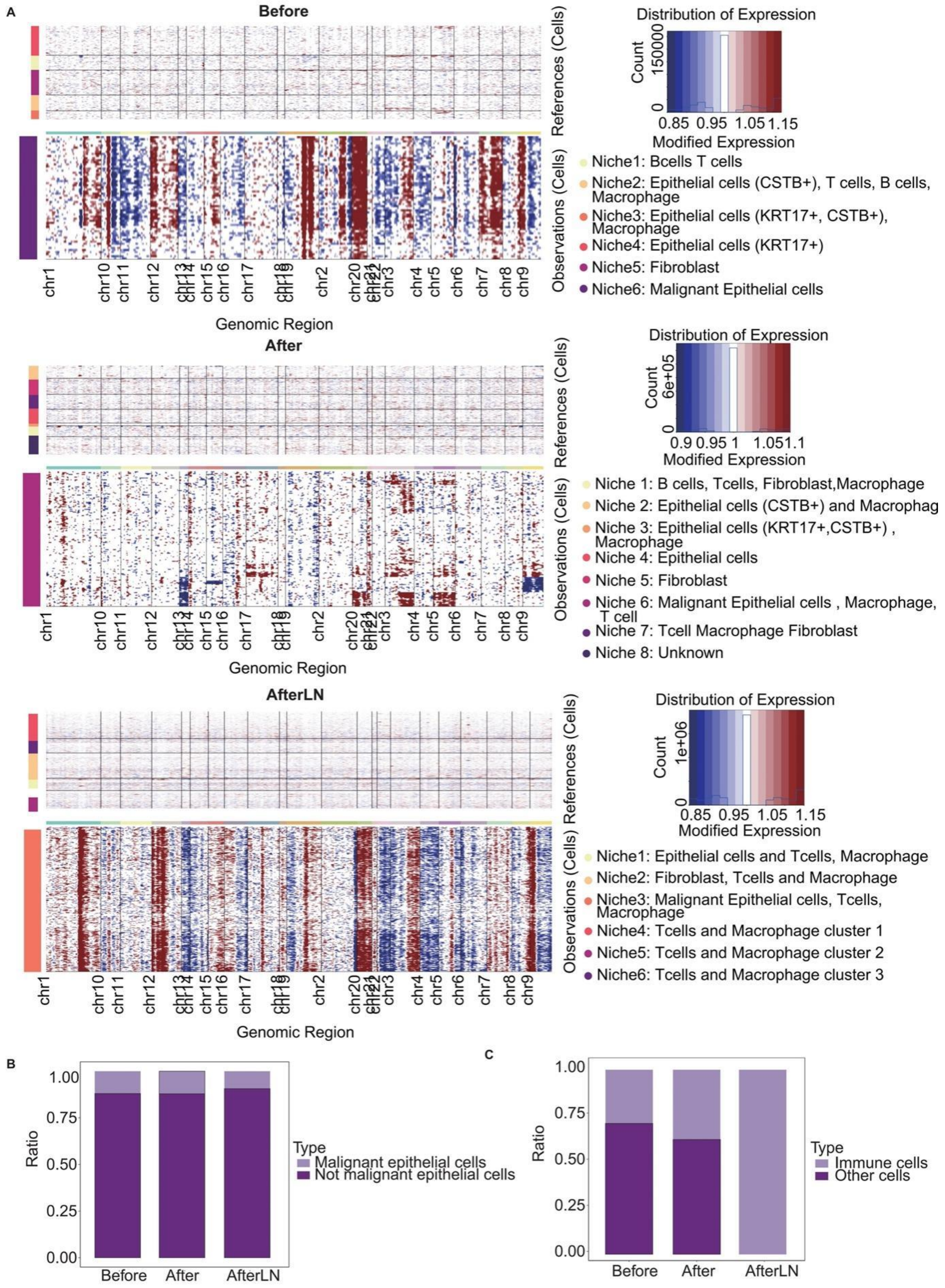

Figure S8. Malignant characterization of spatial data.
(**A**) CNV level of spatial niches in the spatial data. Red represents the amplification. Blue represents the deletion. The color level represents the CNV level. (**B**) The ratio of malignant epithelial cells to nonmalignant epithelial cells in the spatial data. (**C**) The ratio of of immune cells and other cells in the spatial data.

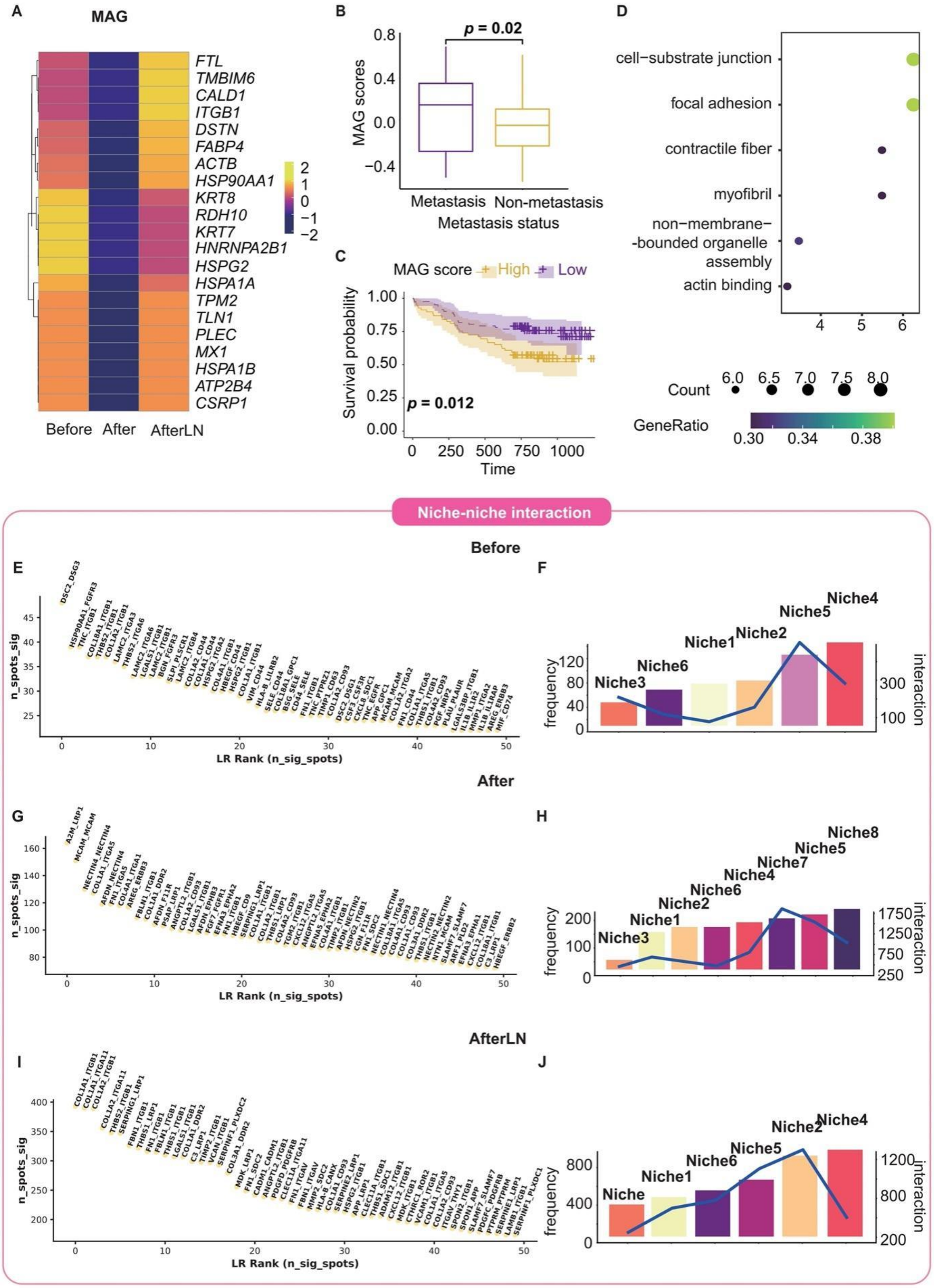

Figure S9. MAGs and ligand–receptor pairs among spatial niches.
(**A**) Heatmap of metastasis-associated genes (MAGs) expressed in all samples. All MAGs are labeled. The colors indicate the expression level. (**B**) Boxplot of MAG scores in patients with

metastasis and patients without metastasis from the PRJCA004501 dataset. P values, two-tailed Wilcoxon test for between-group differences for comparisons. (**C**) Kaplan–Meier curves comparing the overall survival between the MAG-score-high and MAG-score-low groups in the HRA003107 dataset. Curve comparison of p values determined by a two-tailed log-rank (Mantel–Cox) test. (**D**) Gene Ontology pathway enrichment of MAGs. The colors indicate the cell types. (**E, G, I**) The top 50 ligand–receptor pairs with the most spots in the three samples. (**F, H, J**) Cell type frequency and ligand–receptor pair interaction frequency in the three samples.

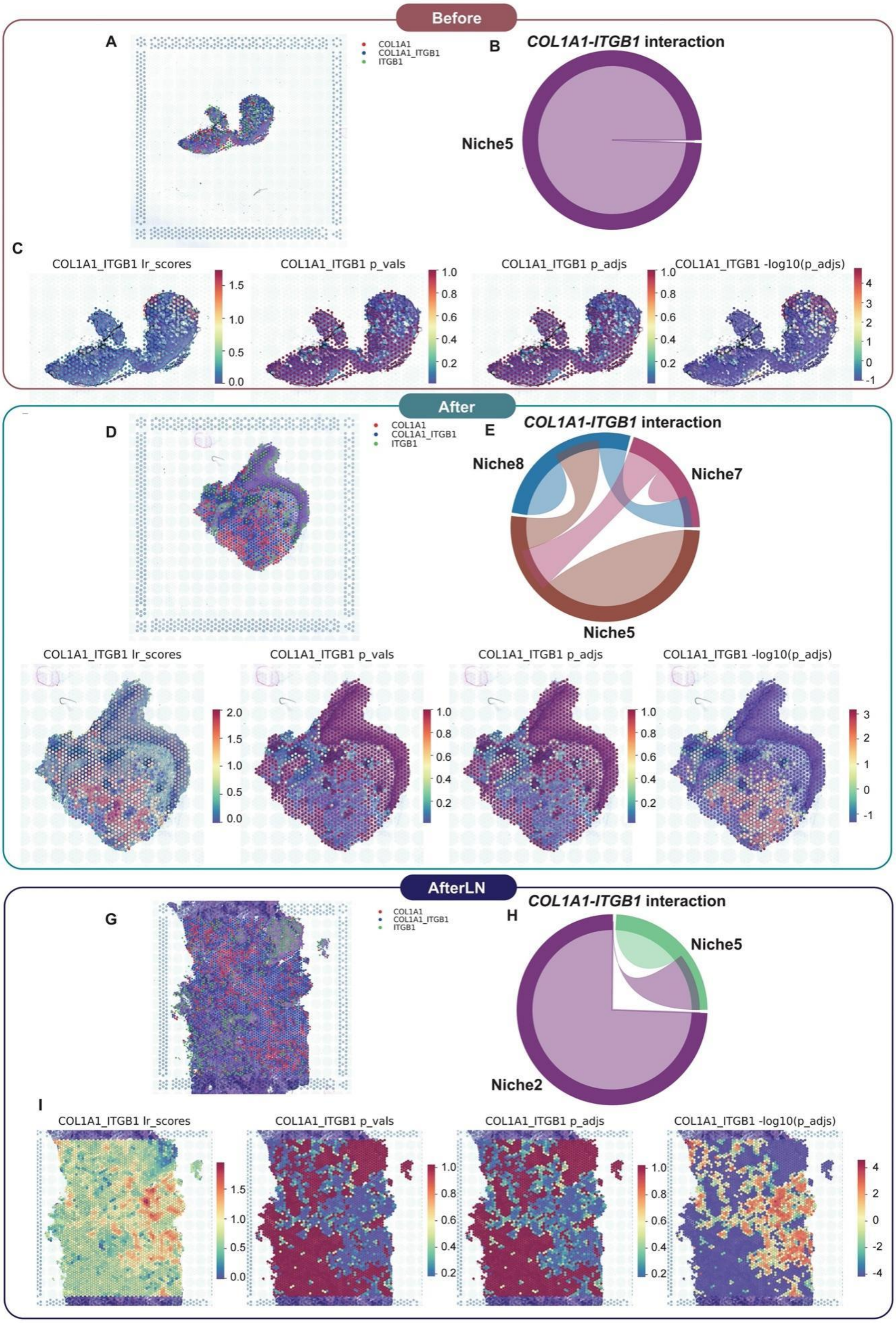

Figure S10. The spatial distribution of the COL1A1-ITGB1 ligand–receptor pair.
(**A, D, G**) The spatial distributions of *COL1A1*, *ITGB1* and COL1A1-ITGB1. Red spots indicate spots where COL1A1 was expressed. Blue spots indicate spots where *COL1A1* and *ITGB1* were

coexpressed. Green spots indicate spots where *ITGB1* was expressed. (**B, E, H**) Cell–cell interaction of the COL1A1–ITGB1 ligand–receptor pair between niches. The colors indicate the niche. The area of each bond indicates the frequency of interaction. (**C, F, I**) Spatial distribution of the ligand–receptor score, p value, p-adjusted value, and -log10 (p adjusted value) of COL1A1-ITGB1 in all samples. The colors indicate the values.

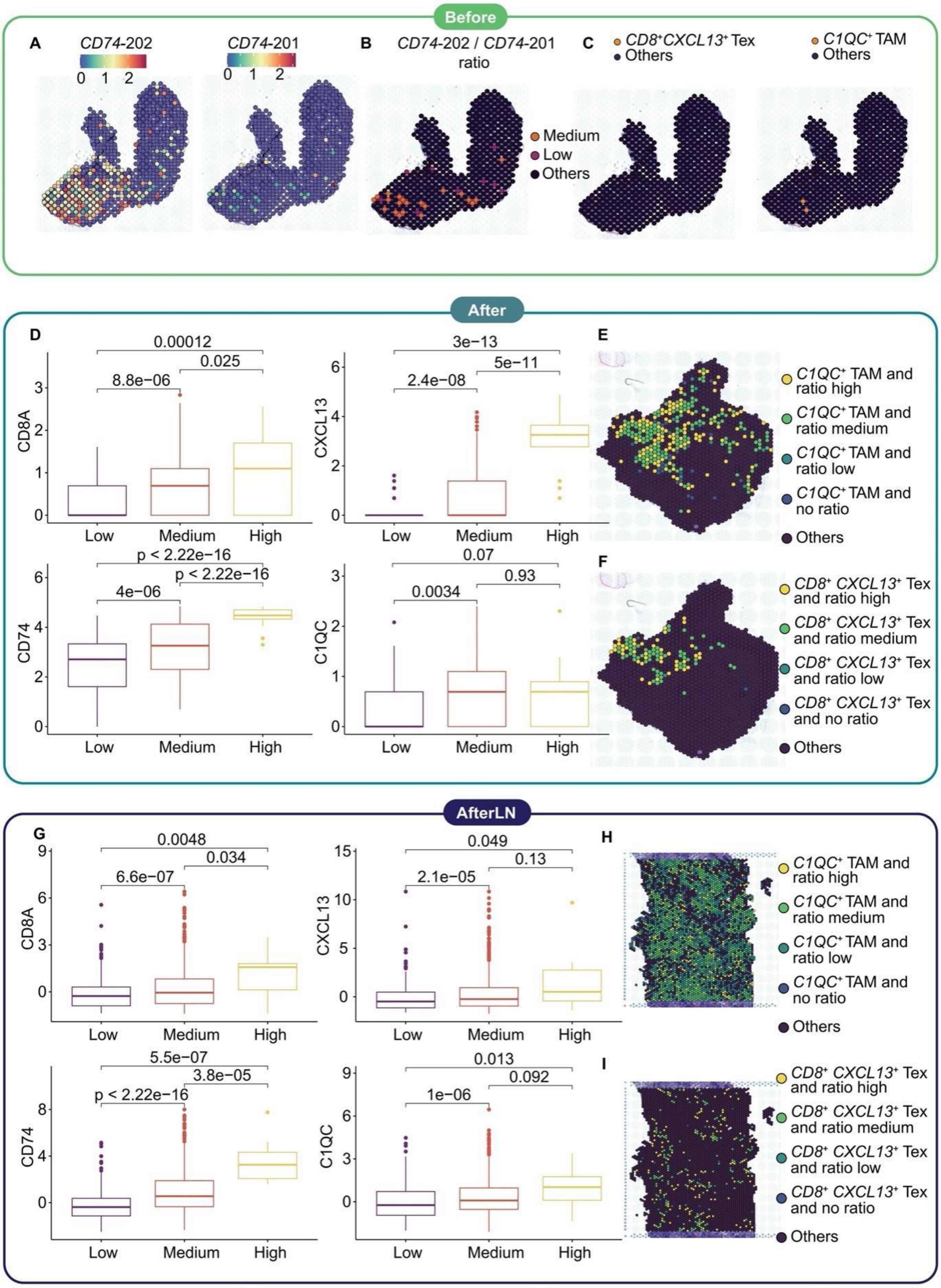

Figure S11. Spatial distribution of the *CD74* isoform ratio and enrichment of *CD8*$^{+}$ *CXCL13*$^{+}$ Tex cells and *C1QC*$^{+}$ TAMs.

(**A**) Spatial distribution of the *CD74*-202 isoform and *CD74*-201 isoform in the Before sample. The colors indicate the expression level. (**B**) Spatial distribution of the ratio of *CD74*-202

isoform expression/*CD74*-201 isoform expression in the Before sample. The colors indicate the ratio of *CD74*-202 isoform expression/*CD74*-201 isoform expression. (**C**) Spatial distribution of *C1QC*$^+$ TAMs, *CD8*$^+$ *CXCL13*$^+$ Tex cells in the Before sample. (**D, G**) Boxplot of the gene expression of *CD8A*, *C1QC*, *CXCL13*, and *CD74* in different *CD74*-202 isoform expression/*CD74*-201 isoform expression ratio groups in the After and AfterLN samples. A two-tailed Wilcoxon test for between-group differences for comparisons was used for P value calculations. (**E, H**) Spatial distribution of spots showing both *C1QC*$^+$ TAMs and *CD74*-202 /*CD74*-201 ratio condition in the After and AfterLN samples. (**F, I**) Spatial distribution of spots showing both *CD8*$^+$ *CXCL13*$^+$ Tex and *CD74*-202 /*CD74*-201 ratio condition in the After and AfterLN samples.

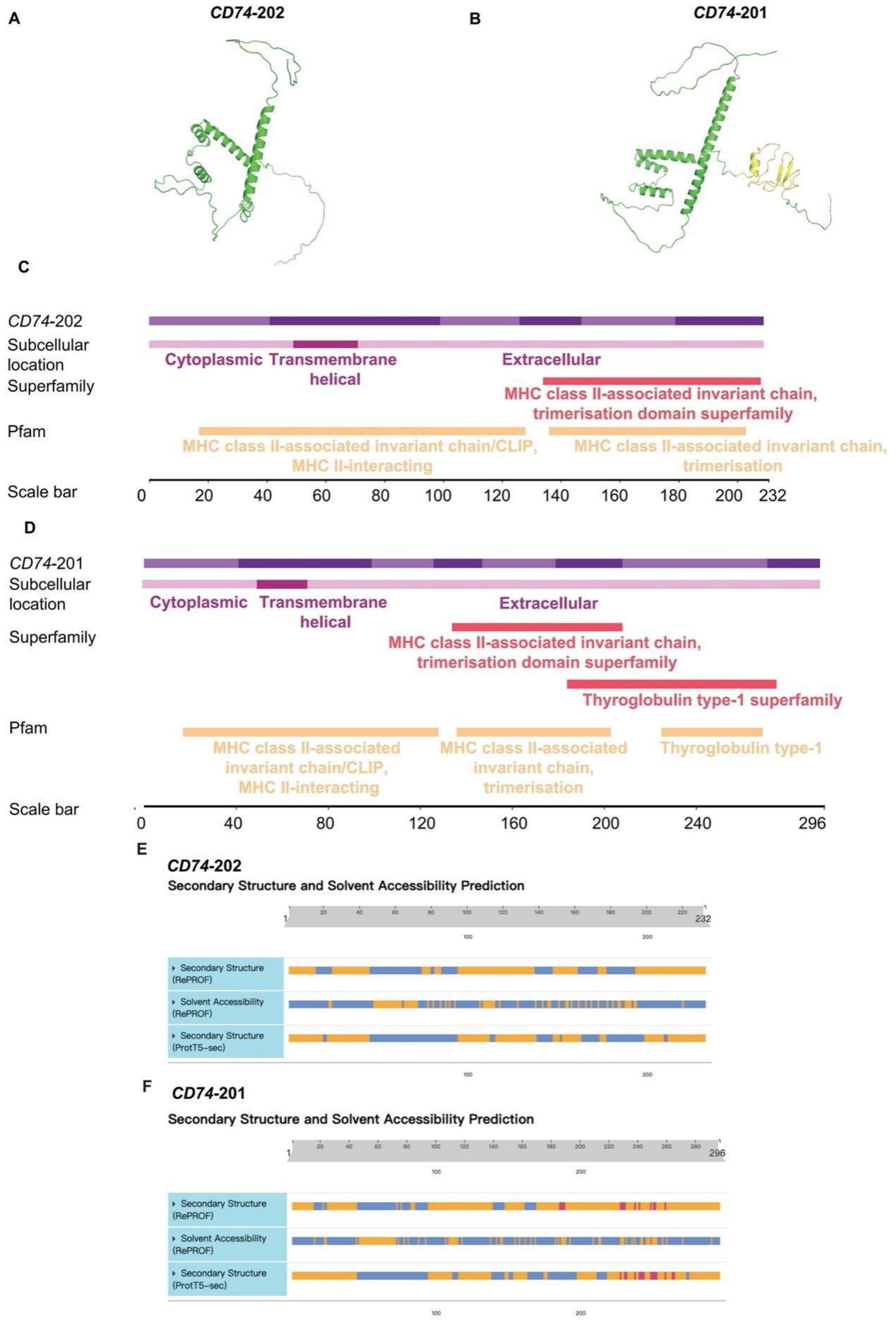

Figure S12. Spatial distribution of the *CD74* isoform ratio and isoform solvent accessibility prediction.
(A-B) 3D structure of the *CD74*-202 isoform and *CD74*-201 isoform predicted by AlphaFold2. (**C-D**) Domains and functional families of the *CD74*-202 isoform and *CD74*-201 isoform. (**E-**

F) Secondary structure and solvent accessibility prediction of the *CD74*-202 isoform and *CD74*-201 isoform.

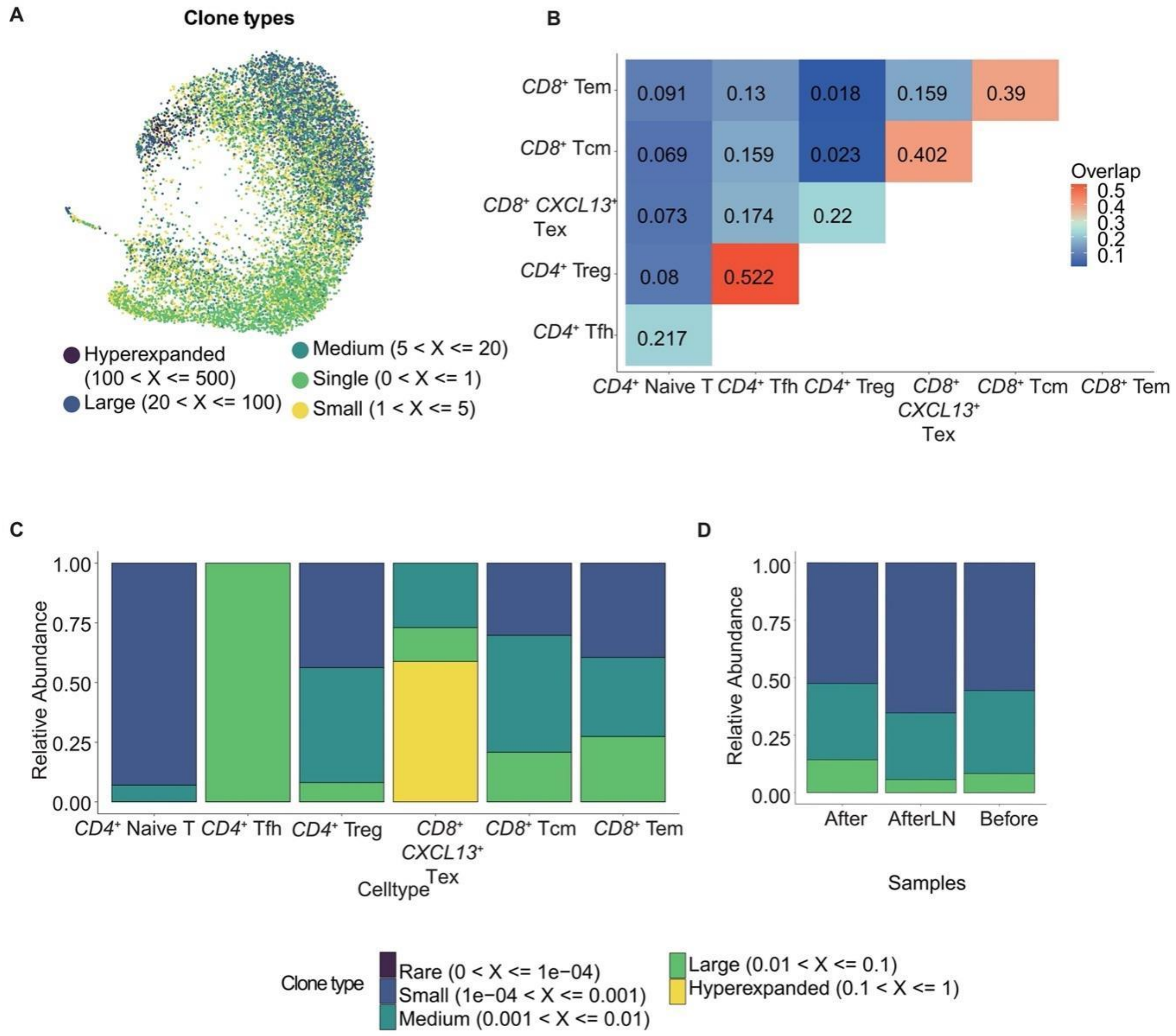

Figure S13. T-cell dynamics in matched single-cell TCR data.
(**A**) TSNE clustering of single-cell transcriptomics data with TCR information annotation. The colors indicate the abundance of the clones. (**B**) Clonal correlation in subclusters of T cells. The colors of the heatmap indicate the correlation level. (**C**) Relative clonal abundance in all subclusters of T cells. (**D**) Relative clonal abundance in all samples.

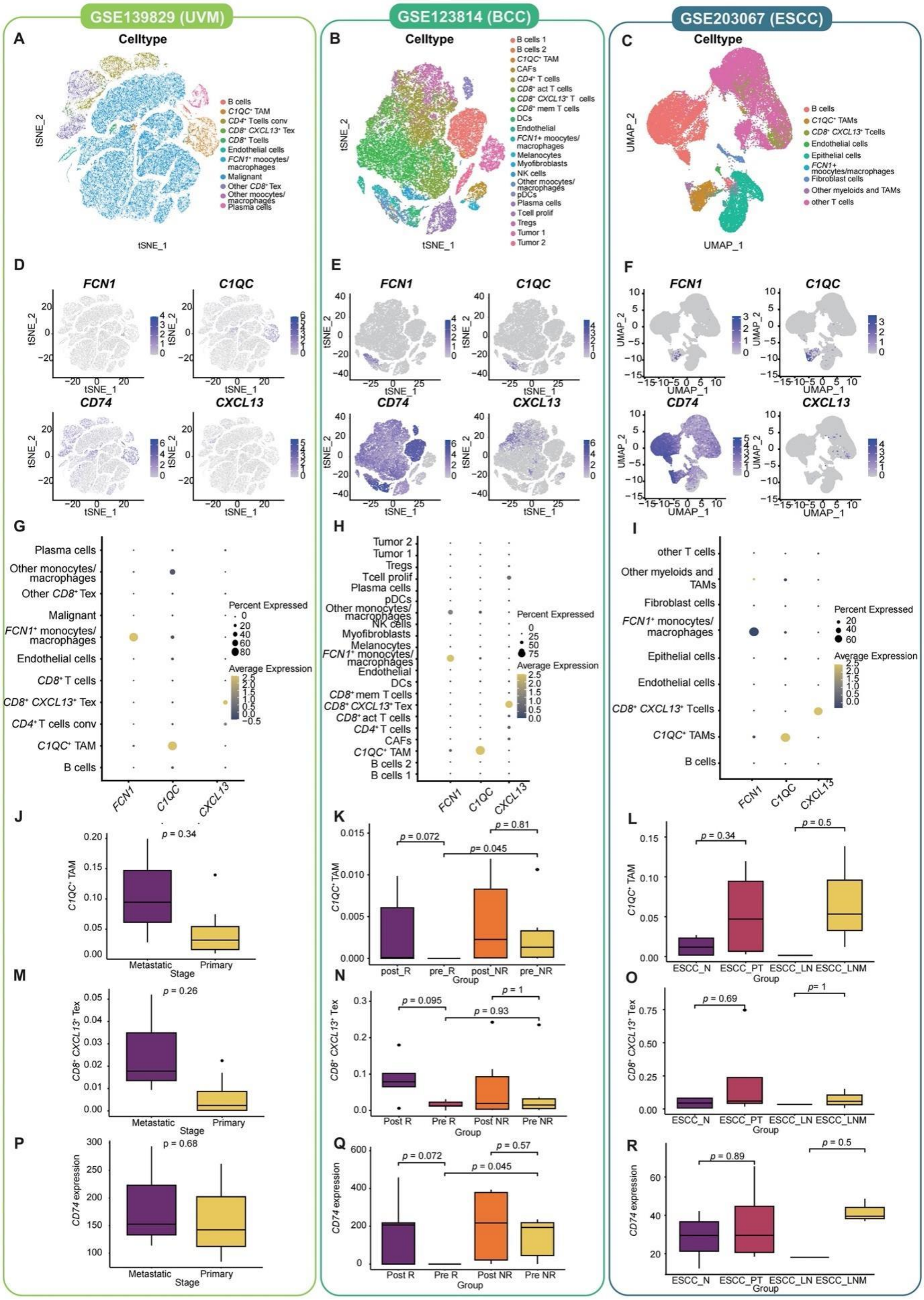

Figure S14. Clustering information for the GSE139829, GSE123814 and GSE203067 datasets.

(**A-C**) TSNE of cell type clustering of single-cell transcriptomics data in the GSE139829, GSE123814 and GSE203067 datasets. The colors indicate the cell type. (**D-F**) *FCN1*, *C1QC*,

CD74 and *CXCL13* gene expression levels in the three datasets. **(G-I)** Dot plot of *FCN1, C1QC* and *CXCL13* gene expression in each cell type in the three datasets. The dot size indicates the percentage of all cells with expression. The colors indicate the average expression level. (**J-L**) Boxplot of the cell ratio of *C1QC*$^{+}$ TAMs in the different groups. The primary stage and metastatic stage were included in the GSE139829 dataset. The groups in the GSE123814 dataset included pretreatment responsive patients, posttreatment responsive patients, pretreatment nonresponsive patients and posttreatment nonresponsive patients. The groups in the GSE203067 dataset included normal mucosa, primary tumor, metastatic LN, and normal LN tissues. (**M-O**) Boxplot of the ratio of *CD8*$^{+}$ *CXCL13*$^{+}$ Tex cells in different groups in the three datasets. A two-tailed t test was used for P value calculations for between-group comparisons. (**P-R**) Boxplot of *CD74* expression in *C1QC*$^{+}$ TAMs in different groups in the three datasets. A two-tailed t test was used for P value calculations for between-group comparisons.

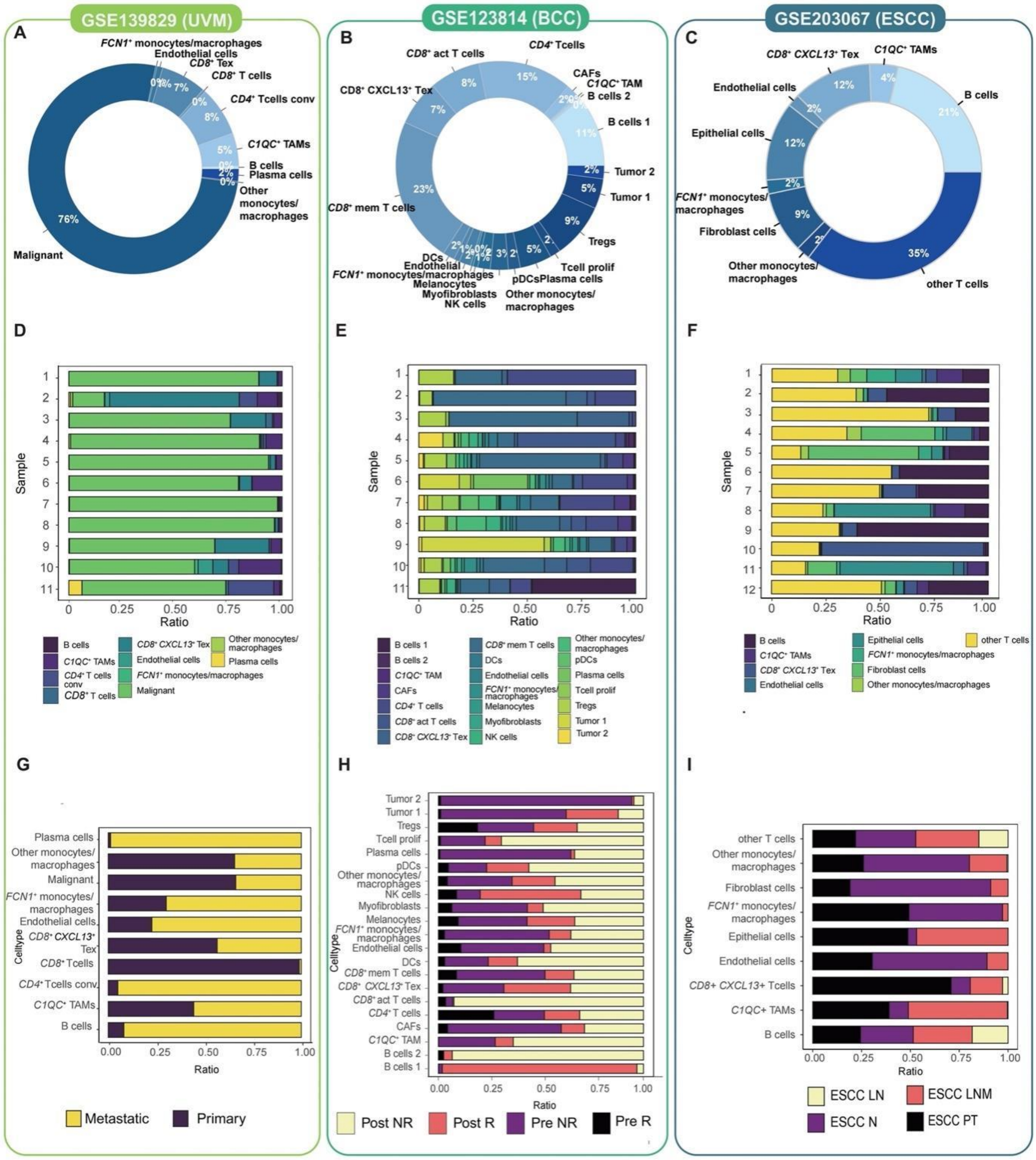

Figure S15. Ratio of cell types to patients in the GSE139829, GSE123814 and GSE203067 datasets.

(**A-C**) Ratio of cell types in the GSE139829, GSE123814 and GSE203067 datasets. (**D-F**) Distribution of cell types in each patient in the GSE139829, GSE123814 and GSE203067

datasets. (**G-I**) Ratio of cells from the metastatic tumor sample and the primary tumor sample in different cell types in the GSE139829, GSE123814 and GSE203067 datasets.

Step	Temperature	Time
1	98°C	00:03:00
2	98°C	00:00:15
3	63°C	00:00:20
4	72°C	00:03:00
5	Go to Step 2, cycle numbers see Supplementary Table S3	
6	72°C	00:05:00
7	4°C	Hold

Table S2. 10X Genomics library preparation cDNA PCR temperature and time.

Targeted Cell Recovery	Total Cycles
<500	13
500-6000	12
>6000	11

Table S3. 10X Genomics library preparation cDNA PCR cycle numbers.
